# Synthesis and Antioxidant Activity of New Catechol Thioethers with the Methylene Linker

**DOI:** 10.3390/molecules27103169

**Published:** 2022-05-16

**Authors:** Ivan V. Smolyaninov, Daria A. Burmistrova, Maxim V. Arsenyev, Maria A. Polovinkina, Nadezhda P. Pomortseva, Georgy K. Fukin, Andrey I. Poddel’sky, Nadezhda T. Berberova

**Affiliations:** 1 Department of Chemistry, Astrakhan State Technical University, 16 Tatisheva Str., 414056 Astrakhan, Russia; burmistrova.da@gmail.com (D.A.B.); ogorodnikova1503@rambler.ru (N.P.P.); nberberova@gmail.com (N.T.B.); 2 G.A. Razuvaev Institute of Organometallic Chemistry, Russian Academy of Sciences, 49 Tropinina Str., 603137 Nizhny Novgorod, Russia; mars@iomc.ras.ru (M.V.A.); gera@iomc.ras.ru (G.K.F.); 3 Toxicology Research Group of Southern Scientific Centre of Russian Academy of Science, 41 Chekhova Str., 344006 Rostov-on-Don, Russia; m.hahaleva@astu.org

**Keywords:** catechol thio-ethers, heterocyclic groups, antioxidants, radical scavenging activity, oxidative damage of the DNA, lipid peroxidation

## Abstract

Novel catechol thio-ethers with different heterocyclic substituents at sulfur atom were prepared by reacting 3,5-di-*tert*-butyl-6-methoxymethylcatechol with functionalized thiols under acidic conditions. A common feature of compounds is a methylene bridge between the catechol ring and thioether group. Two catechols with the thio-ether group, bound directly to the catechol ring, were also considered to assess the effect of the methylene linker on the antioxidant properties. The crystal structures of thio-ethers with benzo-thiazole moieties were established by single-crystal X-ray analysis. The radical scavenging and antioxidant activities were determined using 2,2′-diphenyl-1-picrylhydrazyl radical test, ABTS^∙+^, CUPRAC (TEAC) assays, the reaction with superoxide radical anion generated by xanthine oxidase (NBT assay), the oxidative damage of the DNA, and the process of lipid peroxidation of rat liver (Wistar) homogenates in vitro. Most catechol-thioethers exhibit the antioxidant effect, which varies from mild to moderate depending on the model system. The dual anti/prooxidant activity characterizes compounds with adamantyl or thio-phenol substituent at the sulfur atom. Catechol thio-ethers containing heterocyclic groups (thiazole, thiazoline, benzo-thiazole, benzo-xazole) can be considered effective antioxidants with cytoprotective properties. These compounds can protect molecules of DNA and lipids from the different radical species.

## 1. Introduction

Catechols/hydroquinones and their oxidized forms represent the molecular motifs often found in natural compounds such as polyphenols, flavonoids, alkaloids, amines. These compounds have been considered potential therapeutic agents against inflammation processes, as neuroprotectors, and as COMT inhibitors [1,2,3,4,5] in the case of neurodegenerative diseases. Antioxidants have a presumably therapeutic effect on such diseases. Catechols and their modified analogues possess antibacterial, antitumor, and cytotoxic activities [6,7,8,9]. Besides, catechols exert cyto-protective properties [10], cryo-protective activity under germ cells cryopreservation [11], and can form cryogels [12]. Along with the pronounced biological activity, these compounds are widely used to obtain functionalized materials [13,14,15,16]; they serve as building blocks for coatings design [17], as the basis for organic batteries and redox capacitors [18,19], and as redox-based molecular memory-films [20] due to their ability to accept, store and donate electrons and protons. Researchers consider S-functionalized sterically hindered catechols (quinones) as promising chelating ligands in metal complexes [21,22,23,24,25,26,27], polynuclear compounds and coordination polymers [28,29].

Modulation of the phenols’ and catechols/hydroquinones’ bioactivity, leading to a significant change in their physical properties, reactivity, bioavailability, and pharmacological profile, can be carried out in different ways. One such approach is the introduction of chalcogen atoms into the structure of phenolic compounds. The covalent S-C bonds are often used as a linker to construct polyfunctional molecules which possess diverse biological and pharmacological activity [30]. Sulfur-containing antioxidants attract particular attention [31,32,33,34]. The thiolated derivatives of phenols, tocopherols and ascorbic acid are polyfunctional compounds capable of antioxidant activity and other types of properties [35,36,37,38]. There are thio-ethers with an unsubstituted hydroquinone/catechol core, bonded by sulfide linkers with glutathione or acetylcysteine residues [39,40,41]. Catechols conjugated with bioactive thiols can function as bioavailable antioxidants with less toxicity than parent compounds [42,43]. It was found that the presence of thio-ether linker in the catechol ring or replacement of hydroxyl by thiol group led to an increase in the antioxidant activity of such types of compounds [44,45,46,47].

Now the development of a new synthetic approach to obtain catechol/hydroquinone thio-ethers and the investigation of their structure and properties has become topical [48,49,50,51,52]. The Michael addition reaction of thiols to *ortho*-, *para*-benzoquinones [53,54,55,56] or nucleophilic substitution [57,58] are often applied to synthesize thio-ethers. The application of molecular iodine or diacetoxyiodo-benzene in the reactions of sulfonating agents with cyclohexanones or with masked *o*-benzoquinones leads to thiolated catechols [59,60].

The formation of the C-S bond, playing the role of bridge between redox-active 1,2-, 1,4-dihydroxylbenzene fragments and various functional groups, has attracted interest from the point of view of developing alternative synthesis methods and the search for new properties. Earlier, we carried out *o*-benzoquinone thiolation by different thiols to obtain S-functionalized sterically hindered catechol thio-ethers, and studied antioxidant, radical scavenging activity and redox properties of target compounds [11,61].

**Scheme 1:**
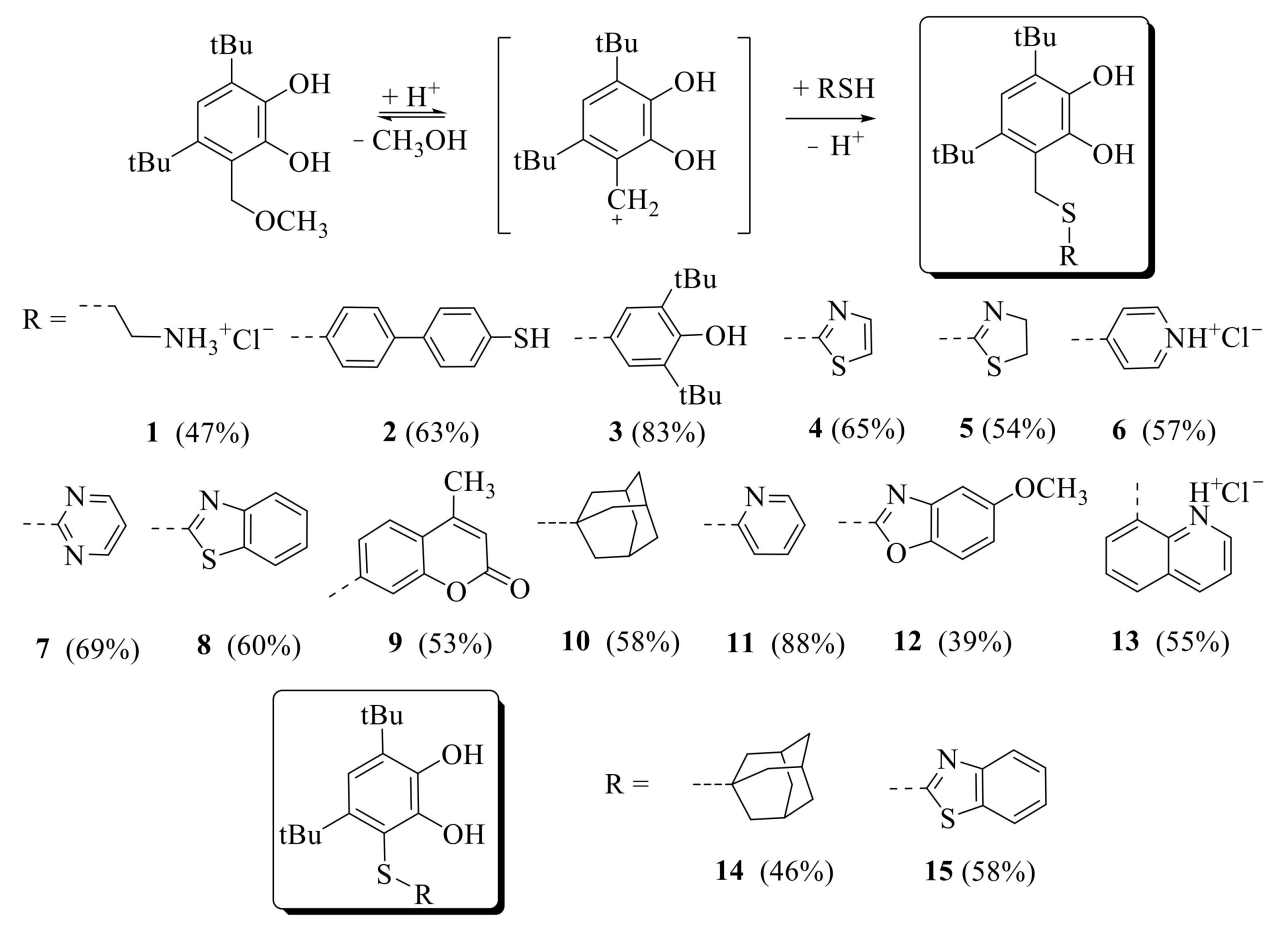
New synthesized catechol thio-ethers **1**–**15**.

In this context, we synthesized a series of thio-ethers with a methylene group isolating catecholic scaffold from a sulfur atom (Scheme 1). These compounds have a sterically hindered catechol moiety playing a primary antioxidant role in the course of electron or hydrogen atom transfer; a thio-ether linker is able to provide secondary antioxidant properties in the terminal (hetero-)aromatic bioactive group. In this paper we discuss the influence of –CH_2_-S- linker and different substituents in the sulfur atom on the radical scavenging, and antioxidant activities of the target compounds. The radical scavenging and antioxidant properties of the compounds were evaluated using the 2,2′-diphenyl-1-picrylhydrazyl radical (DPPH) test, the reaction with 2,2′-azino-bis (3-ethylbenzothiazoline-6-sulfonic acid) radical cation (ABTS^∙+^), Cu(II) ion reducing (CUPRAC) assay in Trolox equivalents (TEAC), the reaction with superoxide radical anion generated by xanthine oxidase (NBT assay), the oxidative damage of the DNA, and the process of lipid peroxidation of rat liver (Wistar) homogenates in vitro.

## 2. Results and Discussion

### 2.1. Synthesis and Characterization

It was previously found that 3,5-di-*tert*-butyl-6-methoxymethylcatechol acts as an alkylating agent in the reactions with activated arenes [62,63] and also interacts with oxygen-, nitrogen-containing nucleophiles [64,65]. In this work, we study the interaction of the above-mentioned catechol with thiols (RSH) mainly containing various biologically active heterocyclic groups (Scheme 1). The proposed reaction mechanism consists of the acid-catalyzed generation of a carbocation, which is attacked by a sulfur-containing nucleophile, followed by the production of the corresponding thio-ethers **1**–**13** (47–83%).

Analogues of **8** and **10** with the thioether group directly bound to the catechol ring, compounds **14** and **15**, were synthesized using the previously described method [11] in order to assess the effect of the methylene linker on the antioxidant properties. The structure of synthesized compounds was confirmed by spectral methods (IR, ^1^H NMR, ^13^C{^1^H} NMR spectroscopy) and elemental analysis. The molecular structures of catechols **8** and **15** in a crystal state were established by single-crystal X-ray analysis.

### 2.2. Crystal Structures

**Figure 1:**
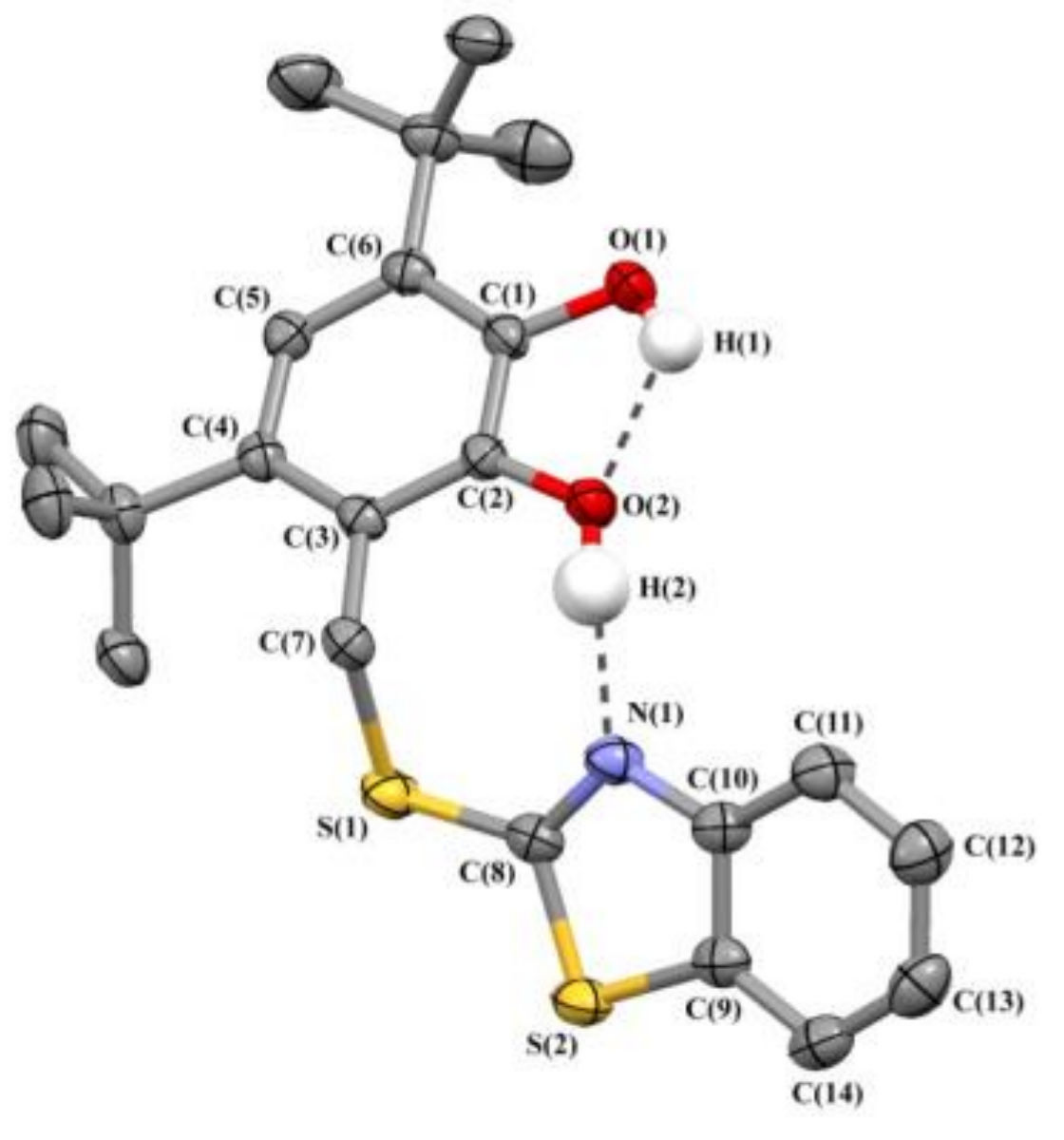
The X-ray structure of **8**. The ellipsoids of 50% probability. The hydrogen atoms except for hydroxy groups are omitted. The selected bonds lengths (Å): O(1)–C(1) 1.377(2), O(2)–C(2) 1.362(2), C(1)–C(2) 1.394(3), C(1)–C(6) 1.380(3), C(2)–C(3) 1.397(2), C(3)–C(4) 1.410(3), C(4)–C(5) 1.392(3), C(5)–C(6) 1.397(3), S(1)–C(7) 1.846(2), S(1)–C(8) 1.735(2), S(2)–C(8) 1.735(2), S(2)–C(9) 1.732(2), N(1)–C(8) 1.299(2), N(1)–C(10) 1.388(3).

The molecular structure of **8** is shown in Figure 1.

The lengths of the oxygen-carbon bonds O(1)–C(1) and O(2)–C(2) as well as the carbon-carbon of the C(1–6) ring are characteristic of catechols [25,65,66,67,68]: O(1)–C(1), 1.377(2) Å; O(2)–C(2), 1.362(2) Å. The carbon-carbon bonds of the six-membered carbon ring C(1–6) average 1.395 ± 0.015 Å and ones are aromatic. The substituent at the C(3) atom is bent from the catechol ring plane by almost 90°: the angle between the C(1,6) and S(1)C(7)C(3) planes is 82.2(1)°. The angle between the C(1–6) catechol plane and the C(9–14)S(2)C(8)N(1) benzothiazole plane is 38.6(1)°. In the crystal, the molecules are packed in such a way that the nitrogen atom N(1) is directed towards the hydroxyl group O(2)H(2). Intramolecular hydrogen bonds are observed in the structure: the O(2)–H(2)...N(1) contact has a distance of 1.82(1) Å, the corresponding O(2)–H(2)–N(1) angle is 172.8 (1)°, and the distance O(1)–H(1)...O(2) is 2.01(1) Å (the corresponding O(1)–H(1)–O(2) angle is 123.8(1)°). These values are typical for intramolecular hydrogen bonding in catechols [68].

**Figure 2:**
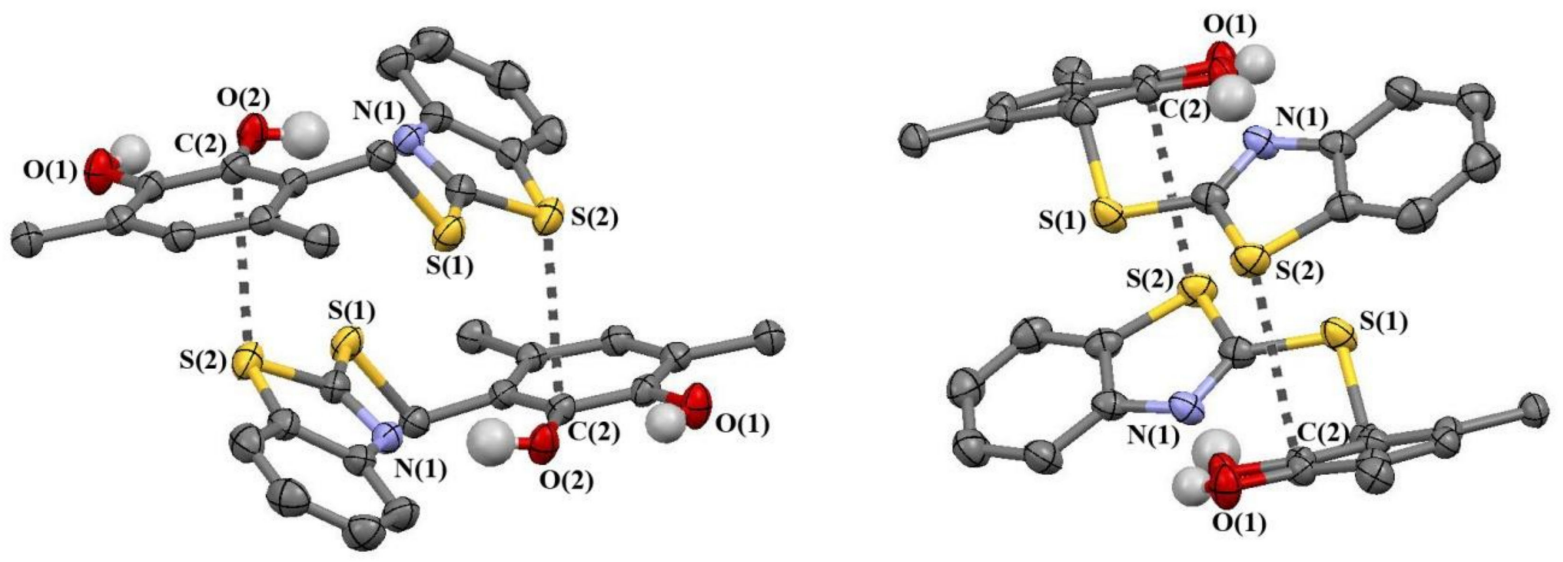
The views on the intermolecular S–C contacts between two neighboring molecules in the crystal of catechol **8** from two different angles. Hydrogen atoms (except hydroxyl) and methyl groups of *tert*-butyl substituents are omitted.

In the crystal, the molecules of **8** are packed in pairs in such a way that short intermolecular contacts S(2)...C(2) of 3.39(1) Å are observed, and their aromatic six-membered carbon rings are arranged in pairs parallel to each other (Figure 2).

**Figure 3:**
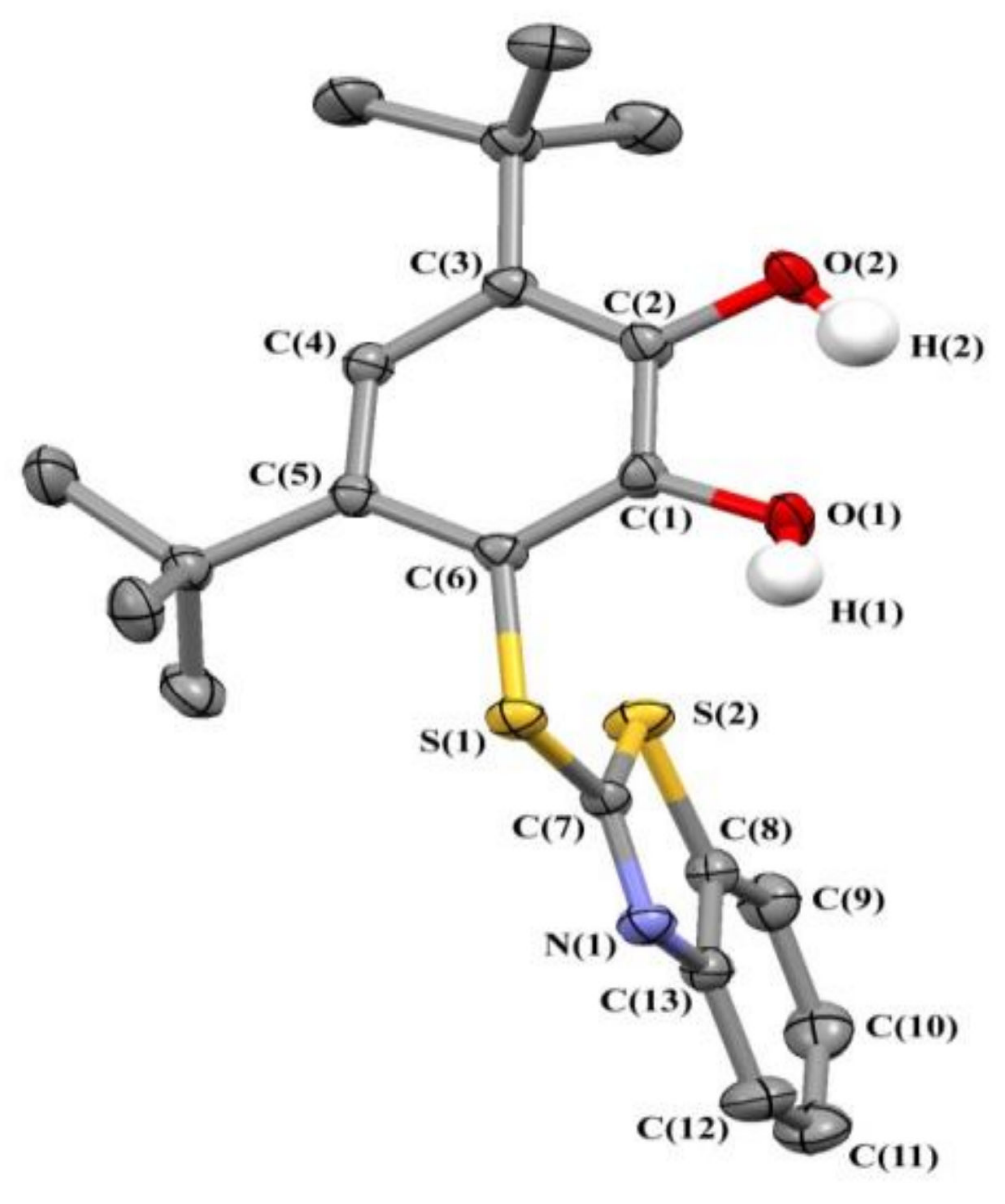
The X-ray structure of **15**. The ellipsoids are of 50% probability. The hydrogen atoms except for hydroxy groups are omitted. The selected bonds lengths (Å): O(1)–C(1) 1.370(2), O(2)–C(2) 1.376(2), C(1)–C(2) 1.397(3), C(1)–C(6) 1.396(3), C(2)–C(3) 1.387(3), C(3)–C(4) 1.403(3), C(4)–C(5) 1.388(3), C(5)–C(6) 1.429(3), C(6)–S(1) 1.781(2), S(1)–C(7) 1.748(2), C(7)–N(1) 1.297(3), C(7)–S(2) 1.744(2), S(2)–C(8) 1.746(2).

The key structural characteristics of **15** also correspond to the parameters typical for catechols, e.g., the O–C bonds are close and ordinary (O(1)–C(1), 1.370(2) Å; O(2)–C(2), 1.376(2) Å), and the carbon-carbon bonds of the six-membered carbon ring C(1–6) are aromatic averaging 1.400 ± 0.029 Å (Figure 3).

**Figure 4:**
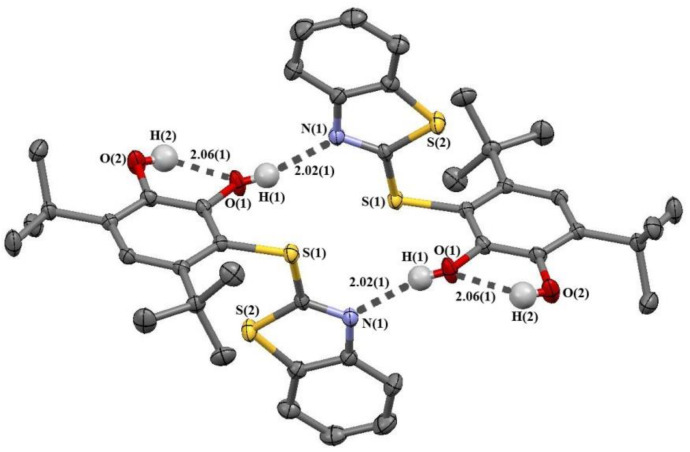
Intra- and intermolecular hydrogen bonds in the crystal of catechol **15**. Hydrogen atoms (except for hydroxyl ones) are omitted.

According to X-ray data, the molecule of **15** is not planar, and the angle between the C(1,6) catechol and N(1)C(7)S(2)C(8–13) benzothiazole planes is 84.4(2)°. The hydroxyl atom H(1) is involved to the intermolecular hydrogen bonding OH...N leading to the pair vise packing of molecules in crystal (Figure 4). The O(2)–H(2)–O(1) angle is 121.9(2)°. Molecules are packed in pairs via intermolecular interactions O(1)–H(1)...N(1) (the corresponding distance is 2.02(1) Å, and the angle O(1)–H(1)–N(1) is 160.6(2)°).

### 2.3. Radical Scavenging and Antioxidant Activity of Target Compounds

We have explored the radical scavenging and antioxidant activity of target compounds with the use of different experimental assays such as the reaction with a diphenylpicrylhydrazyl radical (DPPH), an ABTS^∙+^ radical cation generated by a potassium persulfate, and a CUPRAC test and inhibition process of superoxide radical anion formation by xanthine oxidase (NBT assay). The radical scavenging activity of **1**–**15** in reaction with DPPH varies significantly depending on substituents at the sulfur atom (Table 1). Based on the obtained EC_50_ values in the test with DPPH radical, compounds **2** and **12** containing thiophenol and benzoxazole groups show the maximum activity. The number of converted DPPH molecules (n_DPPH_) is close to 3. Such values indicates the participation of the thiol group in the hydrogen atom abstraction and the electron-donating nature of the substituted benzoxazole.

The n_DPPH_ index for catechols **1**, **3**, **5**, **11**, and **14** reaches an average of 2, as well as for Trolox. Compounds **10**, **13**, and **14** are characterized by large values of time necessary to reach a steady state (TEC_50_) in the series of catechols. The presence of a methylene group between the catechol ring and the thioether group in **10** contributes to a decrease in the efficiency of the antiradical action (AE) compared with **14**. Similar patterns are observed in the test with the radical cation ABTS^∙+^ and CUPRAC_TEAC_ assay. In the case of related compounds **8** and **15** (with or without methylene linker, respectively) containing a benzothiazole fragment, a similar effect was observed only in the reaction with the DPPH radical. In the reaction with ABTS^∙+^, compound **8** is the most active compared to thioether **15** in terms of EC_50_ and TEAC.

Because of the high hydrophobicity, catechol **3** with a phenolic fragment demonstrates a relatively high TEC_50_ value. Deprotonation of the amino group in **1** under the action of 1 eq. of triethylamine leads to a decrease in TEC_50_ to 1 min. Similar behavior is observed for **13** (TEC_50_ decreases to 24 min, and EC_50_ is equal 19 µmol), which may be associated with possible proton-coupled electron transfer (PCET). The EC_50_ value for catechol **4** differs slightly from most of the studied compounds, but at the same time the minimum TEC_50_ is noted.

A feature of compounds **6**–**9** is higher TEC_50_ values compared with that for **4**, while all these catechols have comparable EC_50_ values. The increase of the TEC_50_ value may be associated with the formation of strong hydrogen bonds between the catechol hydroxyl groups and the heterocyclic substituents at the sulfur atom. Catechols containing protonated and free pyridinium groups (**6** and **11**, respectively) have similar TEC_50_ values, but their EC_50_ parameters differ; a more effective antiradical agent is compound **11** with a free pyridine group.

Analysis of the results in terms of AE showed that the compounds could be divided into two groups: the first includes catechols **3**, **6**, **7**, **10** and **14** possessing a moderate activity (AE < 5); the second group consists of the majority of compounds which have a pronounced antiradical efficiency, comparable or higher than that one for Trolox. A feature of these catechols is either the presence of an additional ionizable group that contributes to the possibility of a proton-coupled electron transfer, or a heterocyclic fragment—thiazole, benzo-thiazole, benzoxazole, and coumarin. The presence of a methylene-thioether linker that bonded the catechol fragment with a functional group leads to an increase in the antiradical activity of these compounds compared to 3,5-di-*tert*-butylcatechol (CatH_2_) (AE = 1.3 ± 0.2) [61].

**Scheme 2:**
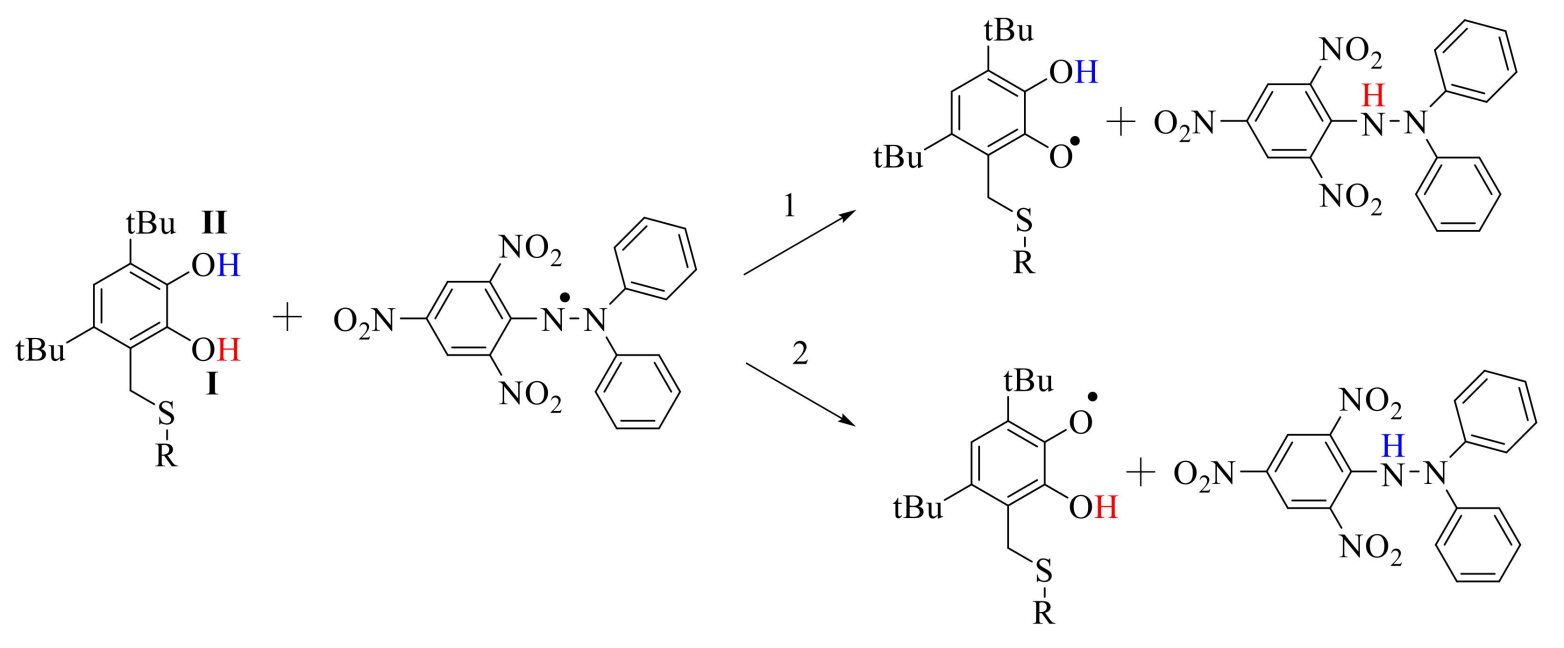
The two alternative sites of DPPH attack in its reaction with the catechol thioether.

Calculations of the bond dissociation energy of O-Н (I, II) (D_O-H_) and thermal effects of the reaction with the DPPH radical (ΔH) were carried out for compounds **1**–**15** to establish the primary centers in the reaction with the radical (Table 2). The value of D_O–H_(I) is, on average, 6.0–10.0 kcal/mol lower than D_O–H_ (II) for most catechols (**2**–**4**,**6**,**7**,**10**–**12**). Therefore, the most likely center of attack for DPPH radical is the OH group in position I of the catechol ring. These data correlate with negative values of ΔH(I) (from −7.6 to −2.8 kcal/mol) upon interaction with DPPH ((1) of Scheme 2).

At the same time, for several compounds (**1**, **5**, and **11**) the calculated HO-bond dissociation energies in both positions of the catechol ring have similar values, and the values of D_O–H_ are lower by 1–9 kcal/mol compared to other thio-ethers. In addition, the measures of ΔH of the hydrogen atom transfer (HAT) in the reaction of DPPH radical with hydroxyl groups are comparable for these substances, and minimum values characterize them. This fact suggests the equally probable participation of both hydroxyls in the HAT reaction. The results are consistent with the number of neutralized molecules of the DPPH (n_DPPH_ ≈ 2). It is possible to compare the ΔH values for **4** and **5**; the aromatization of the five-membered heterocyclic ring leads to a positive ΔH(II) value, an increase in the D_O–H_(II) bond dissociation energy, and a decrease in the number of neutralized radical molecules (n_DPPH_ = 1.16) in the case of **4**.

In the case of thio-ethers **8**, **12**, **13**, and **15** with fused heterocyclic substituents at the sulfur atom, the parameter of D_O-H_ increases for the hydroxyl group in position I, while D_O–H_(II) reduces. The ΔH(II) index has a negative value, which points out a more energetically favorable reaction of the DPPH radical at the HO-group in position II ((2) of Scheme 2). Calculations for compound **8** are in good agreement with the forming hydrogen bonds according to X-ray data. The hydrogen atom of the hydroxyl group in position I interacts with the nitrogen atom of the heterocycle via an intramolecular hydrogen bond. This bond is probably stronger than that between the oxygen atom and the second hydroxyl group. One can assume that similar patterns will also be observed for **12** with the benzoxazole fragment. In the case of **15**, only intermolecular hydrogen bonds are formed between the hydroxyl group in position I of the catechol ring and the nitrogen atom; this can also hinder the abstraction of the hydrogen atom upon interaction with the radical.

Compounds **2** and **3** contain additional groups capable of interacting with the DPPH radical. The calculated dissociation energies of the S–H bond in the thiophenol or of the O–H bond in the phenol group are 77.13 kcal/mol and 83.33, respectively. The ΔH value of the reaction of the DPPH radical with catechol **2**, accompanied by hydrogen atom abstraction from the thiol group, is negative (−4.12 kcal/mol). At the same time, this parameter for phenolic fragment of compound **3** is positive (2.07 kcal/mol). Therefore, in the case of catechol **2**, the thiophenol and catechol groups can participate in the reaction with the DPPH radical, which increases the number of converted radical molecules to three. The catechol fragment can act as the primary center of attack for compound **3** in the reaction with a stable radical.

To evaluate the antioxidant capacity, we applied the ABTS assay, which is also one of the most widely used methods. Unlike the DPPH method, where the realization of both HAT and PCET mechanisms is possible, the ABTS test belongs to the group of analyses relating to the electron transfer and characterizing the electronic capacity of molecules. In CUPRAC assay, bis-(neo-cuproine)copper(II) chelate-type complex, (Neo)_2_Cu(II), also acts as an outer sphere electron transfer agent [69]. In most cases, the IC_50_ values for catechols in the reaction with the ABTS^∙+^ agree with similar data for the DPPH radical. Catechols **8** and **12** with heterocyclic groups have minimal IC_50_ values. This result is consistent with the TEAC value, which is almost three times higher than the Trolox data (Table 1). The TEAC index is close to 2 for catechol **9**. In the case of **3**, this parameter exceeds such value; the fact suggests the participation of the phenol group in the ABTS^∙+^ reduction reaction. However, this compound tends to auto-oxidation in order to form a dimer that explains the understated results of activity in the assay.

The TEAC values for **4**, **5**, **7**, **11**, and **13** (with heterocyclic substituents at the sulfur atom) range from 1.17 to 1.65. The TEAC data for **3**, **8**, and **12** are comparable to those reported for dehydroquercetin, apigenin, curcumin, and gallic acid (TEAC = 2.52–2.89) [70,71]. In the ABTS test, the radical scavenging activity of catechols also exceeds the results obtained for hydroxy-tyrosol and their esters (0.77) [72]. For compounds **7** and **15**, such activity is close to the activity of caffeic or ferulic acids [70]. The radical scavenging properties of thio-ethers **1**, **6**, **10**, and **15** are comparable to those of Trolox, while other catechols in our series have more pronounced activity. The appearance of differences in the reactivity of catechols in the reaction with two synthetic radicals is due to the acceptor capacity of radicals. Based on electrochemical experiments, the electron transfer agents can be ordered according to their reduction potential: (Neo)_2_Cu(II) (−0.58 V) < DPPH (0.25 V) < ABTS^∙+^ (0.55 V) (GC-electrode, MeCN, vs. Ag/AgCl/KCl sat.). The most active oxidizing agent is ABTS^∙+^, which can potentially oxidize catechol and electron donor benzoxazole, benzothiazole, phenol, and thiophenol groups.

The method for determining the electronic capacity of an antioxidant, which uses a milder oxidant, is a test with a bis-(neo-cuproine)copper(II) complex. The TEAC values vary from 0.71 to 0.96 for most of the catechols. In case of catechol **1**, the results obtained are consistent with greater activity in the DPPH test, which is caused by the possibility of partial ionization of the cysteamine hydrochloride residue. Autoxidation of the thiol group in compound **2** occurs in the CUPRAC test during the incubation time (30 min), so the TEAC value is close to that for CatH_2_ (0.72 ± 0.05) [73]. Catechols with pyrimidine **7** and pyridine **11** groups have similar TEAC values, and these parameters exceed the results obtained for Trolox. The activity of compound **6** in the presence of 1 eq. triethylamine increases (1.19 ± 0.6) and is close to the data for catechol **11**. Compound **12** with a methoxybenzoxazole moiety possesses the highest antioxidant capacity, while catechol **13** is slightly inferior to it in terms of TEAC. In the case of both compounds, there is a good correlation between the three methods for determining antiradical activity.

### 2.4. Inhibition of Superoxide Formation by Xanthine Oxidase (NBT Assay)

The superoxide radical anion is included in the pool of the reactive oxygen species (ROS), forming in various metabolic processes and ensuring the functioning of many enzymatic systems. Excessive production of O_2_^∙−^ can lead to negative consequences, causing redox balance disturbance, biomacromolecules destruction and, accordingly, oxidative stress induction. Previously, we have shown that catechol thio-ethers can act as neutralizers of O_2_^∙−^ generated under electrochemical conditions [55]. In the present study, we use the NBT assay to evaluate superoxide radical anion scavenging activity. Superoxide radical anion is generated by the xanthine-xanthine oxidase. This intermediate can be reduced by nitro blue tetrazolium (NBT) to the blue colored (560 nm) formazan. Compounds that inhibit the formazan formation can be considered scavengers of superoxide radical anion. 

At a concentration of 100 µmol, highly hydrophobic molecules **3**, **10**, and **14** did not show pronounced activity in this test due to their low solubility in the buffer solution. At the same time, depending on the degree of inhibition (%), the stages of O_2_^∙−^ formation catechols can be arranged in the following order: **13** (5.02 ± 0.07) < **1** (27.08 ± 0.07) < **11** (36.5 ± 0.04) < **9** (36.85 ± 0.06) < **2** (38.49 ± 0.05) < **8** (50.69 ± 0.09) < **6** (52.76 ± 0.07) < **15** (67.10 ± 0.02) < **12** (67.82 ± 0.03) < **5** (68.41 ± 0.02) < **7** (89.28 ± 0.01) < **4** (96.93 ± 0.07). Salts **1** and **13** play the role of inefficient superoxide radical anion scavengers. Compounds **9** and **2** with coumarin and thiophenol groups showed comparable moderate activity, as well as catechols **8** and **6** with heterocyclic substituents at the sulfur atom. We have observed a similar activity for catechol thio-ethers with hydrophobic hydrocarbon groups under electrochemical conditions in acetonitrile [55]. However, for most compounds, the established IC_50_ values are equal to ≥100 µmol (Table 1). Catechols **4**, **5**, **7**, **12**, and **15** should be noted among the target compounds since these thio-ethers revealed a pronounced superoxide scavenging activity. Compounds **4** and **5** have the lowest IC_50_ values in this test, confirming their high neutralizing properties against the superoxide anion radical. Substances **7**, **15** and **12** are the more active O_2_^∙−^ scavengers than Trolox.

Earlier, Kamperman et al. [13] has shown that the interaction of catechols with O_2_^∙−^ occurs with a formation of HOO-radical, H_2_O_2_ and *o*-benzoquinones. These intermediates can promote the oxidative damage of biomacromolecules. On the one hand, the considered catechols contain a thio-ether linker which behaves as a secondary antioxidant capable of decomposing H_2_O_2_, ROOR [74]. On the other hand, we have previously shown that sterically hindered *o*-benzoquinones formed during the redox cycle have the ability to neutralize O_2_^∙−^ and form stable *o*-semiquinone anion radicals, as well as inhibit the lipid peroxidation process [75]. Recently it has been found that the reaction of 3,5-di-*tert*-butyl-*o*-benzoquinone with a hydroperoxyl radical is accompanied by hydrogen atom abstraction and leads to 2-hydroxyphenoxyl radical and oxygen [76].

In the case of catechol thio-ethers, the presence of the sterically hindered catechol group causes the ability to donate a hydrogen atom, while the thioether linker can be characterized by antiperoxidative properties; the combination of these two structural fragments with heterocyclic scaffolds suggests the appearance of pronounced protective activity during oxidative damage of biomacromolecules such as DNA or lipids.

### 2.5. Promoted Oxidation of DNA and Lipid Peroxidation

**Figure 5:**
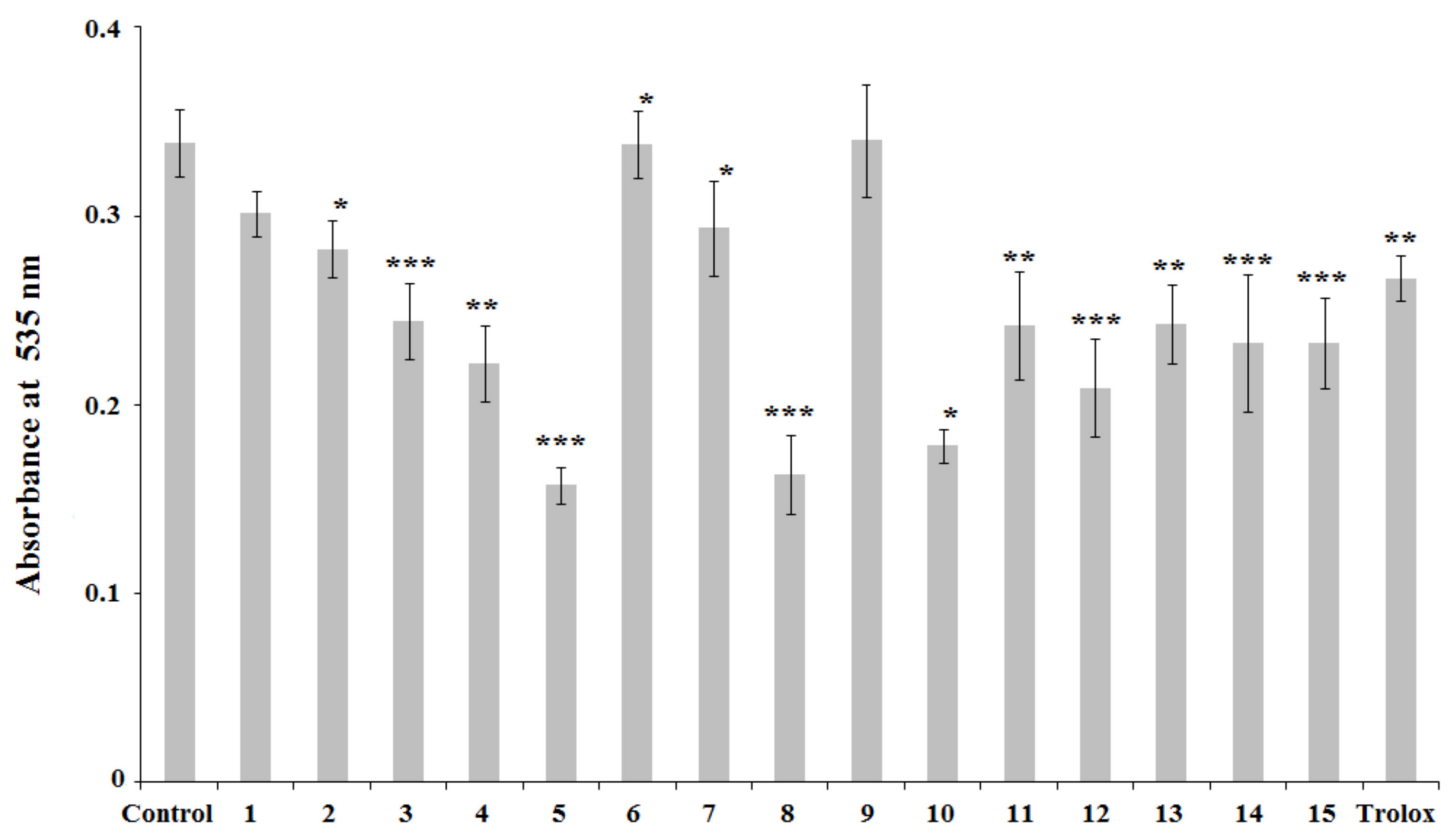
The variety of the absorbance of TBARS in the DNA oxidation (2.0 mg∙mL^−1^) induced by 40 mM AAPH in the presence of **1**–**15**, Trolox, and without additives (Control). The results are expressed as mean ± standard deviation (* *p* < 0.05; ** *p* < 0.005; *** *p* < 0.001).

The study of the antioxidant properties of catechol thio-ethers **1**–**15** was continued by the experiments on promoted oxidative damage of the DNA molecules by 2,2′-azobis(2-amidinopropane) dihydrochloride (AAPH). The initiator decomposes to radical intermediates that induce DNA damage. This process leads to degradation products that form a colored complex with thio-barbituric acid (TBARS). The target compounds can donate hydrogen atoms, neutralizing peroxyl radicals and preventing the development of DNA oxidative damage. The AAPH induced oxidation of DNA was carried out with additives of catechol thio-ethers **1**–**15** and Trolox at a concentration equal to 50 µmol. The absorbance of TBARS in UV-vis spectra was measured after the oxidation during 150 min in comparison with that in the blank experiment (Control) (Figure 5).

We have found that experiments with catechols **6** and **9** reveal absorbance indicators comparable with the control experiment (Figure 5). They do not have a marked antioxidant effect. In the presence of catechols **1**, **2**, and **7**, low efficiency of the DNA inhibition is observed (11–16%). In the case of compound **1**, methylene linker contributes to a decrease in antioxidant activity compared to the previously studied catechol, where a thioether group was bound directly to the catechol ring [11]. In general, for most compounds, an antioxidant effect is observed, in contrast to CatH_2_, which has a weak promoting effect [73]. Deprotonation of compound **6** with triethylamine and the subsequent administration into the incubation medium promotes a decrease in the value absorbance by 27%. The antioxidant activity of the deprotonated form of **6** becomes comparable to the data observed for catechol **11**. Similar changes occurred for substance **1**: the antioxidant activity increased from 11 to 21%. However, for compound **13** in the presence of a base, no significant influence on the value of absorbance was observed. Catechols **3**, **4**, **11**–**15** are proper a moderate inhibition efficiency (28–38%) and their antioxidant activity exceeds the Trolox results (21%). An analogue of compound **3** without -CH_2_- linker was also characterized by a moderate activity in this assay [61]. The greatest lowering in TBARS absorbance occurred in the presence of compounds **5**, **8**, and **10** containing thiazoline (53%), benzo-thiazole (52%), and adamantyl (47%) substituents at the sulfur atom, respectively. The change of the thiazoline group by the thiazole group in **4** leads to a decrease in inhibitory activity. In contrast to the DPPH test, ABTS and CUPRAC assays, for **10** and **14**, an inverse relationship is observed: the antioxidant activity is higher when a methylene linker group belongs between the catechol ring and the sulfur atom. At the same time, the data on inhibition of the DNA oxidative damage and the results of ABTS and CUPRAC tests become more consistent for catechols **8** and **15**. Compound **8** possesses an expressed inhibitory effect.

**Figure 6:**
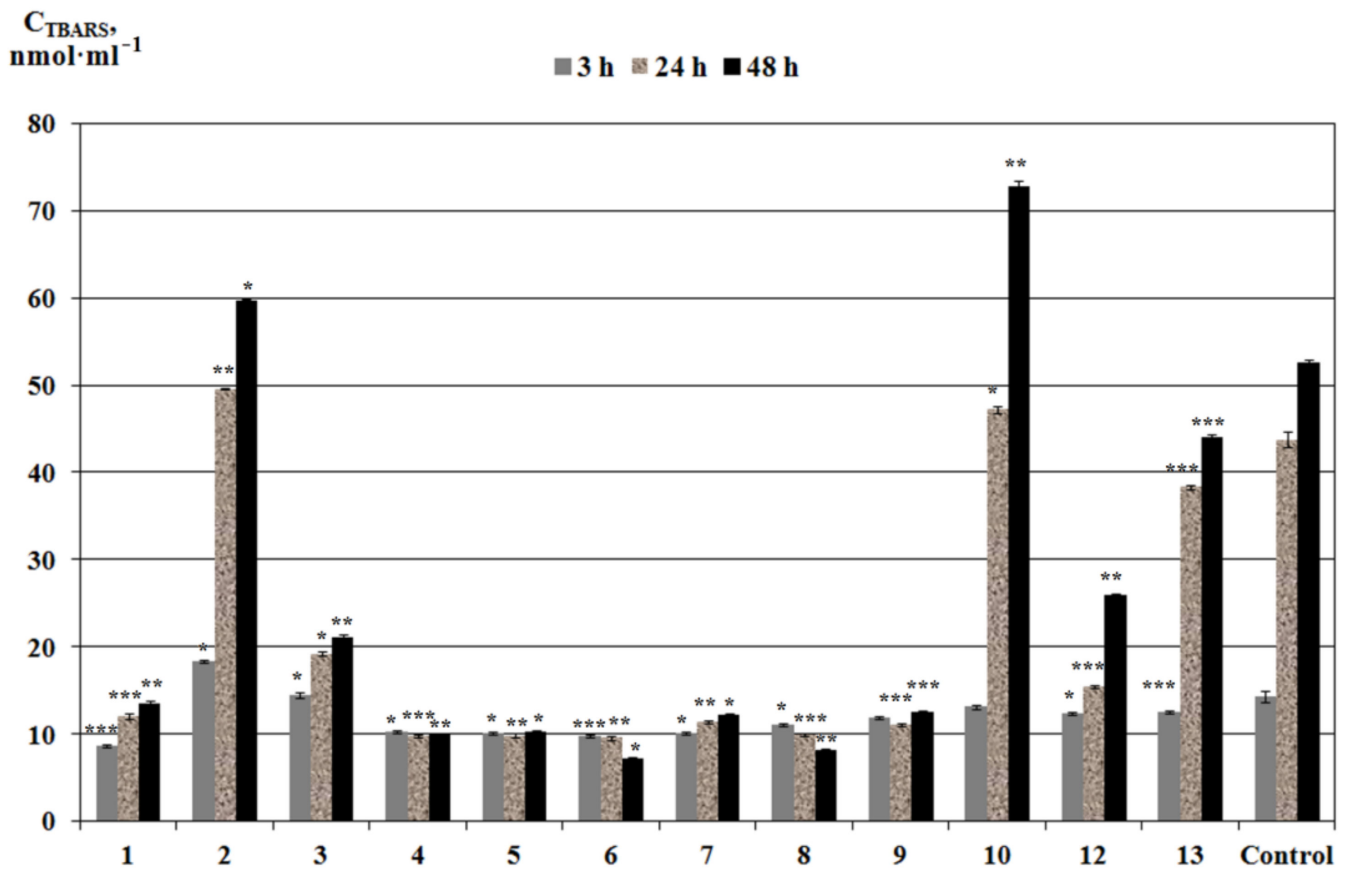
The level of TBARS in rat liver homogenates (Wistar) in vitro, in the presence of compounds **1**–**10**, **12**, **13**, and without additives (Control) during the incubation 3, 24, 48 h (concentration of compounds in the reaction medium is 0.1 mM). The results are expressed as mean ± standard deviation (* *p* < 0.05; ** *p* < 0.005; *** *p* < 0.001).

Earlier we have shown that several catechol thio-ethers inhibit the lipid peroxidation (LP) process in vitro [11,61]. It is interesting to determine the influence of CH_2_-S-linker in the structure of target compounds on the lipid peroxidation reaction of the rat (Wistar) liver homogenate as a non-enzymatic process induced by Fe(II) ions (in vitro). The degree of lipid peroxidation of the rat liver homogenates was assessed by the accumulation of TBARS products [77]. The samples of the rat brain homogenates were divided as follows: one control (blank experiment) and homogenates with additives of catechols. TBARS concentrations were determined by measuring the absorbance of the solution at 535 nm using UV-vis spectroscopy (Figure 6).

Thioethers **1**, **3**, **4**–**9**, **12**, and **13** possess an antioxidant activity reducing the concentration of TBARS compared to the control experiment. The inhibition effect for these compounds at the initial moment (3 h) allows for a decrease in the TBARS amounts by 8–40%. These data are consistent with the previously obtained results for catechol thio-ethers with alcohol, carboxyl groups, or an acetylcysteine fragment [11]. Compound **1** exhibits a pronounced antioxidant activity (3 h), lowering the content of TBARS by 40%, in contrast to its analogue without a methylene group, which was found to be inactive [11].

In this experiment, we increased the incubation time to 48 h to determine the long-term effects of the substances on the lipid peroxidation process. The TBARS concentration in the experiment in the presence of catechol **3** (with a fragment of sterically hindered phenol group) is comparable to the control experiment at the starting point (3 h). The inhibition action begins to show only at remote stages of the LP. Compounds **12** and **13** initially had a weak antioxidant activity (12–13%), and this trend continued throughout the experiment for compound **13**. In contrast, catechol **12** has been characterized with more marked antioxidant properties over incubation. In the case of additives of compounds **1**, **3**, and **12**, the change in the TBARS content occurs regularly with an increase in those for the control experiment. At the same time, the inhibitory effect of catechols is enhanced by 56–74%.

Compounds **2** and **10** reveal an evident promoting effect on the LP process, increasing the concentration of TBARS. Thioether **2** is characterized by an autooxidation reaction with the formation of a disulfide. In reactions with lipid hydroperoxides generated in the course of the LP reaction, the catechol fragment remains active in both compounds; this moiety can oxidize to *o*-semiquinone anion radical and then *o*-benzoquinone. In the presence of iron ions, these species can promote lipid peroxidation. A feature of compounds **4**–**9** is the presence of heterocyclic fragments in their structure. These substances maintain an almost constant concentration of TBARS during the incubation time. In the case of thio-ethers **6** and **8** with fragments of pyridinium salt and benzothiazole, the TBARS amount gradually decreases during the experiment. At the same time, the antioxidant effect reaches the maximum values of 86 and 84%, accordingly. An increase in the antioxidant activity of the deprotonated forms of **1** and **6** (1 eq. of triethylamine) occurs only at the primary moment (3 h) by 8 and 14%, compared with the initial results. The inhibitory influence of the deprotonated form of compound **1** on LP becomes similar to that of the starting material with a duration time increase. In the presence of the deprotonated form of catechol **6**, the TBARS value increases by 10–12%, which indicates a decreasing inhibitory effect. Analysis of the data for this group of compounds indicates their prolonged action and effective stabilization of the LP process when the concentration of lipid peroxidation products increases significantly with time for the control experiment.

The combination of data on the radical scavenging activity of catechols in the reactions with synthetic radicals, superoxide radical anion, and bis-(neo-cuproine)copper(II) complex, and the determination of the effect on the processes promoting damage of macro-biomolecules (DNA, lipids) allows identification of three target groups of compounds. Catechols **1**, **3**, **6**, **7**, **9**, **11**, and **13**–**15** comprise the most numerous group with a predominantly antioxidant effect, which varies from mild to moderate depending on the model system. Compounds **10** and **2** with an adamantyl or thio-phenol substituent at the sulfur atom form the second group of substances with dual anti/prooxidant activity. The absence in this series of thio-ethers with heterocyclic residues suggests that activity mainly relating to the realization of the catechol/*o*-benzoquinone redox cycle leads to reactive oxygen species generation. The third group combines thio-ethers **4**, **5**, **8**, and **12** with heterocyclic moieties such as thiazole, thiazoline, benzo-thiazole, and benzoxazole. These substances can be considered effective antioxidants with cytoprotective properties, capable of protecting biomacromolecules from active radical particles formed during the oxidative stress.

## 3. Materials and Methods

### 3.1. General Remarks

All starting reagents were commercially available: 3,5-di-*tert*-butyl-*o*-benzoquinone (99%, Alfa Aesar, Kandel, Germany), 8-mercaptoquinoline hydrochloride (96%, TCI), 2,2′-azobis(2-amidinopropane) dihydrochloride (AAPH) (97%, Aldrich, Munich, Germany), 2-mercaptoethylamine hydrochloride (98%, Acros Organics, Geel, Belgium), biphenyl-4,4′-dithiol (95%, Sigma Aldrich, Taufkirchen, Germany), 2,6-di-*tert*-butyl-4-mercaptophenol (97%, Sigma Aldrich), 2-mercaptothiazoline (98%, ABCR, Karlsruhe, Germany), 2-mercaptothiazole (98%, Acros Organics), 4-mercaptopyridine (95%, Sigma Aldrich), 2-mercaptopyrimidine (98%, Sigma Aldrich), 2-mercaptobenzothiazole (98%, Acros Organics), 7-mercapto-4-methylcoumarine (≥97%, Sigma Aldrich), 1-adamantanethiol (95%, Sigma Aldrich), 2-mercaptopyridine (99%, Sigma Aldrich), 5-methoxybenzoxazole-2-thiol (97%, Sigma Aldrich), 2,2′-azino-bis(3-ethylbenzothiazoline-6-sulfonic acid) (≥98%, TCI, Tokyo, Japan), neocuproine (98%, Sigma Aldrich) 2,2-diphenyl-1-picrylhydrazyl (98%, Aldrich), thio-barbituric acid (≥98%, Sigma-Aldrich), deoxyribonucleic acid sodium salt (DNA) from salmon testes (Sigma), phosphate buffered saline (PBS) pH 7.4 (Sigma), xanthine oxidase from bovine (Sigma-Aldrich, grade IV), nitroblue tetrazolium (90%, Alfa Aesar, Kandel, Germany), xanthine (≥99%, Sigma-Aldrich), bovine serum albumin (≥96%, Sigma-Aldrich), 3,5-di-*tert*-butylcatechol (98%, Aldrich), 6-hydroxy-2,5,7,8-tetramethylchromane-2-carboxylic acid (Trolox) (97%, Aldrich), trichloroacetic acid (≥99%, Sigma-Aldrich), Neo-cuproine (2,9-dimethyl-1,10-phenanthroline) (99%, Aldrich), EDTA, xanthine, bovine serum albumin, nitroblue tetrazolium, and xanthine oxidase (0.04 MU) (Aldrich) and used without further purification in the synthesis of the target compounds and biological tests. 3,5-Di-*tert*-butyl-6-methoxymethylcatechol was synthesized by known method [78].

The IR spectra were recorded on an FSM-1201 FT-IR spectrometer in KBr pellets. The NMR spectra were measured in CDCl_3_ or DMSO-d_6_ on a Bruker Avance DPX-200, Bruker Avance HD 400 spectrometers with a frequency of 200 MHz (^1^Н) or 400 MHz (^1^Н) and 50 MHz (^13^C) or 100 MHz (^13^C) using Me_4_Si as an internal standard. The chemical shift values are given in ppm with the reference to solvent and the coupling constants (J) are given in Hz. The elemental analysis was carried out on a Euro EA 3000 (C, H, N) and Analytik Jena multi EA 5000 (C, S, N, Cl) elemental analyzers. Mass spectra (HRMS) for compounds **5** and **14** were recorded on a Bruker UHR-TOF Maxis^TM^ Impact mass spectrometer (ESI). Quantum-chemical calculations were performed by the DFT method at the B3LYP/6-31++G(d,p) level of theory using HyperChem 8.0.8 software (Würzburg, Germany).

The UV-VIS spectra were recorded with a SF-104 spectrophotometer (in a range of 350–900 nm) or Multiskan Sky microplate spectrophotometer (Thermo Scientific). Electrochemical studies were carried out using VERSASTAT-3 potentiostate (PAR) in three-electrode mode. The stationary glassy carbon (*d* = 2 mm) disk was used as working electrode; the auxiliary electrode was a platinum-flag electrode. The reference electrode was Ag/AgCl/KCl (sat.) with watertight diaphragm. All measurements were carried out under argon. The samples were dissolved in the pre-deaerated solvent. The scan rate (ν) was 200 mV∙s^−1^. The supporting electrolyte 0.1 M Bu_4_NClO_4_ (99%, electrochemical grade, Fluka) was dried in vacuum (48 h) at 50 °C. The concentration of DPPH, ABTS^∙+^, bis-(neocuproine)copper(II) complex was 1–3 mmol.

### 3.2. X-ray Diffraction Studies

The X-ray diffraction data were collected on a Bruker D8 Quest (for **8**) and Agilent Xcalibur EOS (for **15**) diffractometers (Mo-Kα radiation, ω-scan technique, λ = 0.71073 Å) at 298K. The intensity data were integrated by SAINT [79] and CrysAlisPro [80] programs. SADABS [81] (**8**) and SCALE3 ABSPACK [79] (**15**) were used to perform area-detector scaling and absorption corrections. The structures were solved by dual-space [82] method and were refined on F^2^ using all reflections with the SHELXTL package [83]. All non-hydrogen atoms were refined anisotropically. The H atoms were placed in calculated positions and refined in the “riding-rotating model”. The H atoms of OH groups in **8** and **15** were localized and refined objectively. The details of crystallographic, collection and refinement data for **8** and **15** are presented in Table S1. CCDC-2166872 (**8**) and 2166873 (**15**) contain the supplementary crystallographic data for this paper. These data can be obtained free of charge from The Cambridge Crystallographic Data Centre via ccdc.cam.ac.uk/data_request/cif (accessed on 13 May 2022).

### 3.3. Synthesis of Catechol Thioethers **1**–**15**

The reaction of 3.5-di-*tert*-butyl-6-methoxymethylcatechol (0.54 g, 2 mmol) with thiols (2 mmol) was carried out in acetic acid (10 mL) under an inert atmosphere (argon) with stirring for 6 h at 60 °C. After the reaction, 20 mL of water was added to the solution; the resulting precipitate was filtered off, dried in a vacuum, and recrystallized from acetonitrile. In case of catechol 6 the solution of concentrated hydrochloric acid (2 mL) was added in water solution. The formed precipitate of pyridinium salt was filtered off, dried in a vacuum. Compound **3** was previously prepared [25]. 

3-((2-Aminoethylthio)methyl)-4,6-di-*tert*-butylcatechol hydrochloride (**1**). Yield 0.326 g (47%). Beige powder with m.p. 196–198 °C. IR (KBr, ν/cm^−1^): 3394, 3208, 2991, 2954, 2910, 2869, 2714, 2609, 1604, 1587, 1530, 1486, 1418, 1390, 1364, 1286, 1257, 1194. ^1^H NMR (200 MHz, DMSO-d_6_, δ, ppm): 1.32 (s, 9 H, tBu), 1.35 (s, 9 H, tBu), 2.85 (t, ^3^J(H,H) = 6.9 Hz, 2 H, SCH_2_CH_2_NH_3_), 3.07 (t, ^3^J(H,H) = 6.9 Hz, 2 H, SCH_2_CH_2_NH_3_), 4.02 (s, 2 H, CH_2_), 5.39 (s, 5 H, OH, -NH_3_), 6.69 (s, 1 H, C_6_H_1_ arom.). ^13^C NMR (50 MHz, DMSO-d_6_, δ, ppm): 29.56, 30.03, 30.72, 32.24, 34.55, 35.29, 114.49, 122.21, 133.77, 137.53, 142.64, 145.62. Calcd. for C_17_H_30_ClNO_2_S (%): C, 58.68; H, 8.69; Cl, 10.19; N, 4.03; S, 9.21. Found (%): C, 58.70; H, 8.85; Cl, 10.01; N, 4.13; S, 9.19.

3-((4′-Mercaptobiphenyl-4-ylthio)methyl)-4,6-di-*tert*-butylcatechol (**2**). Yield 0.570 g (63%). Beige powder with m.p. 150–152 °C. IR (KBr, ν/cm^−1^): 3529, 3353, 3083, 3025, 2954, 2913, 2873, 2551,1591, 1480, 1421, 1394, 1365, 1350, 1293, 1232, 1198. ^1^H NMR (200 MHz, CDCl_3_, δ, ppm): 1.44 (s, 9H, tBu), 1.45 (s, 9H, tBu), 4.54 (s, 2H, CH_2_), 6.04 (br.s, 1H, OH), 6.43 (br.s, 1H, OH), 6.99 (s, 1H, C_6_H_1_ arom.), 7.30-7.80 (m, 8H, arom.). ^13^C NMR (50 MHz, CDCl_3_, δ, ppm): 29.26, 31.20, 34.80, 35.38, 36.74, 117.16, 118.44, 127.55, 127.70, 129.86, 130.52, 130.60, 134.30, 134.49, 138.69, 139.17, 142.89, 143.27. Calcd. for C_27_H_32_O_2_S_2_ (%): C, 71.64; H, 7.13; S, 14.16. Found (%): C, 71.59; H, 7.28; S, 14.10.

3-((Thiazol-2-ylthio)methyl)-4,6-di-*tert*-butylcatechol (**4**). Yield 0.456 g (65%). Red-brown powder with m.p. 181–183 °C. IR (KBr, ν/cm^−1^): 3482, 3123, 3106, 2954, 2910, 2873, 1537, 1482, 1420, 1382, 1363, 1339, 1285, 1246, 1220, 1200, 1165. ^1^H NMR (200 MHz, CDCl_3_, δ, ppm): 1.41 (br.s, 18H, tBu), 5.61 (s, 2H, CH_2_), 5.65 (br.s, 1H, OH), 6.24 (br.s, 1H, OH), 6.55 (d, ^3^J(H,H) = 4.7 Hz, 1H, CH), 6.85 (d, ^3^J(H,H) = 4.7 Hz, 1H, CH), 6.97 (s, 1H, C_6_H_1_ arom.). ^13^C NMR (50 MHz, CDCl_3_, δ, ppm): 29.58, 32.31, 34.89, 35.70, 47.22, 111.27, 116.67, 117.15, 130.70, 135.66, 139.62, 141.73, 144.29, 186.98. Calcd. for C_18_H_25_NO_2_S_2_ (%): C, 61.50; H, 7.17; N, 3.98; S, 18.24. Found (%): C, 61.41; H, 7.29; N, 4.05; S, 18.22.

3-((4,5-Dihydrothiazol-2-ylthio)methyl)-4,6-di-*tert*-butylcatechol (**5**). Yield 0.380 g (54%). Beige-brown powder with m.p. 176–178 °C. IR (KBr, ν/cm^−1^): 3478, 3221, 3039, 2976, 2950, 2909, 2872, 1621, 1571, 1496, 1449, 1415, 1367, 1348, 1308, 1286, 1237, 1200. ^1^H NMR (400 MHz, CDCl_3_, δ, ppm): 1.40 (s, 9H, tBu), 1.41 (s, 9H, tBu), 3.25 (t, ^3^J(H,H) = 7.5 Hz, 2H, CH_2_), 3.64 (t, ^3^J(H,H) = 7.5 Hz, 2H, CH_2_), 4.82 (s, 2H, CH_2_S), 6.03 (s, 1H, OH), 6.88 (s, 1H, C_6_H_1_ arom.), 7.33 (s, 1H, OH). ^13^C NMR (100 MHz, CDCl_3_, δ, ppm): 25.63, 29.35, 32.70, 34.85, 35.69, 42.69, 48.60, 116.00, 116.70, 134.06, 138.34, 142.07, 143.75, 176.71. HR-MS: Found *m*/*z*: 352.1415 [M+H]^+^. C_18_H_26_NO_2_S_2_. Calcd. *m*/*z*: 352.1410.

3-((Pyridin-4-ylthio)methyl)-4,6-di-*tert*-butylcatechol hydrochloride (or 4-(4,6-Di-*tert*-butyl-2,3-dihydroxybenzylthio)pyridinium chloride) (**6**). Yield 0.430 g (57%). White crystalline powder with m.p. 195–197 °C. IR (KBr, ν/cm^−1^): 3326, 3194, 3127, 3086, 3049, 2995, 2957, 2907, 2876, 2744, 1627, 1593, 1492, 1480, 1421, 1386, 1288, 1254, 1230,1202. ^1^H NMR (400 MHz, DMSO-d_6_, δ, ppm): 1.34 and 1.36 (both s, each 9 H, tBu), 3.52 (br.s, 1 H, NH), 4.62 (s, 2H, CH_2_), 6.80 (s, 1H, C_6_H_1_ arom.), 7.96 (d, ^3^J(H,H) = 6.9 Hz, 2H, C_5_H_4_N), 8.30 (br.s, 1H, OH), 8.61 (d, ^3^J(H,H) = 6.9 Hz, 2H, C_5_H_4_N), 8.79 (br.s, 1H, OH). ^13^C NMR (100 MHz, DMSO-d_6_, δ, ppm): 29.48, 30.96, 31.98, 34.75, 35.32, 115.28, 117.60, 121.73, 135.77, 138.67, 140.03, 141.28, 142.62, 146.00. Calcd. for C_20_H_28_ClNO_2_S (%): C, 62.89; H, 7.39; Cl, 9.28; N, 3.67; S, 8.39. Found (%): C, 62.85; H, 7.50; Cl, 9.34; N, 3.73; S, 8.35.

3-((Pyrimidin-2-ylthio)methyl)-4,6-di-*tert*-butylcatechol (**7**). Yield 0.478 g (69%). Yellow powder with m.p. 133–135 °C. IR (KBr, ν/cm^−1^): 3302, 3127, 3086, 3035, 2961, 2913, 2873, 1571, 1553, 1437, 1421, 1387, 1292, 1202. ^1^H NMR (200 MHz, CDCl_3_, δ, ppm): 1.41 (s, 9H, tBu), 1.46 (s, 9H, tBu), 4.72 (s, 2H, CH_2_), 6.24 (br.s, 1H, OH), 6.94 (s, 1H, C_6_H_1_ arom.), 7.08 (t, ^3^J(H,H) = 4.9 Hz, 1H, C_4_H_3_N_2_ arom.), 8.60 (d, ^3^J(H,H) = 4.9 Hz, 2H, C_4_H_3_N_2_ arom.), 10.61 (s, 1H, OH). ^13^C NMR (50 MHz, CDCl_3_, δ, ppm): 29.38, 30.04, 32.82, 34.84, 35.94, 116.43, 116.64, 120.41, 133.48, 138.23, 142.76, 143.55, 157.24, 173.36. Calcd. for C_19_H_26_N_2_O_2_S (%): C, 65.86; H, 7.56; N, 8.09; S, 9.25. Found (%): C, 65.94; H, 7.70; N, 8.14; S, 9.35. 

3-((Benzo[d]thiazol-2-ylthio)methyl)-4,6-di-*tert*-butylcatechol (**8**). Yield 0.481 g (60%). Beige crystals with m.p. 158–160 °C. IR (KBr, ν/cm^−1^): 3472, 3059, 3025, 2961, 2910, 2866, 1570, 1466, 1428, 1392, 1380, 1360, 1312, 1289, 1267, 1246, 1220, 1200. ^1^H NMR (200 MHz, CDCl_3_, δ, ppm): 1.42 (s, 9H, tBu), 1.47 (s, 9H, tBu), 4.94 (s, 2H, CH_2_), 6.49 (s, 1H, OH), 6.97 (s, 1H, C_6_H_1_ arom.), 7.35 (td, ^3^J(H,H) = 7.6 Hz, ^4^J(H,H) = 1.0 Hz, 1H, C_6_H_4_ arom.), 7.49 (td, ^3^J(H,H) = 7.6 Hz, ^4^J(H,H) = 1.0 Hz, 1H, C_6_H_4_ arom.), 7.74 (d, ^3^J(H,H) = 7.8 Hz, 1H, C_6_H_4_ arom.), 7.94 (d, ^3^J(H,H) = 7.9 Hz, 1H, C_6_H_4_ arom.), 11.32 (br.s, 1H, OH). ^13^C NMR (50 MHz, CDCl_3_, δ, ppm): 29.38, 32.92, 33.01, 34.89, 36.10, 116.75, 120.50, 121.24, 124.93, 126.60, 133.85, 134.88, 138.06, 143.50, 143.64, 150.96, 171.00. Calcd. for C_22_H_27_NO_2_S_2_ (%): C, 65.80; H, 6.78; N, 3.49; S, 15.97. Found (%): C, 65.83; H,6.94; N, 3.52; S, 16.05.

7-(4,6-Di-*tert*-butyl-2,3-dihydroxybenzylthio)-4-methyl-2H-chromen-2-one (**9**). Yield 0.452 g (53%). Yellow powder with m.p. 206–208 °C. IR (KBr, ν/cm^−1^): 3512, 3418, 3093, 2991, 2961, 2910, 2873, 1710, 1685, 1604, 1598, 1559, 1540, 1493, 1418, 1398, 1387, 1372, 1362, 1327, 1295, 1247, 1230. ^1^H NMR (400 MHz, CDCl_3_, δ, ppm): 1.39 (s, 9H, tBu), 1.42 (s, 9H, tBu), 2.41 (s, 3H, CH_3_), 4.52 (s, 2H, SCH_2_), 5.90 (s, 1H, OH), 5.95 (s, 1H, OH), 6.25 (s, 1H, CH cum.), 6.97 (s, 1H, C_6_H_1_ arom.), 7.25 (d, ^3^J_(H,H)_ = 8.3 Hz, 1H, C_6_H_3_ arom.), 7.31 (s, 1H, C_6_H_3_ arom.), 7.52 (d, ^3^J_(H,H)_ = 8.3 Hz, 1H, C_6_H_3_ arom.). ^13^C NMR (100 MHz, CDCl_3_, δ, ppm): 18.60, 29.40, 32.18, 32.86, 34.97, 35.68, 114.53, 115.30, 117.37, 117.50, 118.03, 123.65, 124.87, 134.67, 138.98, 141.59, 142.72, 143.21, 151.98, 153.85, 160.36. Calcd. for C_25_H_30_O_4_S (%): C, 70.39; H, 7.09; S, 7.52. Found (%): C, 70.44; H, 7.26; S, 7.55.

3-(Adamantane-1-yl-thio)methyl)-4,6-di-*tert*-butylcatechol (**10**). Yield 0.467 g (58%). White powder with m.p. 188–190 °C. IR (KBr, ν/cm^−1^): 3485, 3283, 2991, 2954, 2907, 2869, 1487, 1469, 1396, 1359, 1292, 1263, 1240, 1207, 1168. ^1^H NMR (CDCl_3_, 200 MHz, δ, ppm): 1.39 (s, 9H, tBu), 1.41 (s, 9H, tBu), 1.70-1.86 (m, 6H, CH_2Ad_), 1.97-2.08 (m, 6H, CH_2Ad_), 2.13 (br.s, 3H, CH_Ad_), 4.04 (s, 2H, CH_2_S), 5.99 (s, 1H, OH), 6.87 (s, 1H, OH), 6.91 (s, 1H, C_6_H_1_ arom.). ^13^C ЯМР (50 MHz, CDCl_3_, δ, ppm): 25.01, 29.42, 29.69, 32.16, 34.88, 35.55, 36.24, 43.12, 45.69, 116.72, 119.00, 133.37, 138.36, 142.75, 143.09. Calcd. for C_25_H_38_O_2_S (%): C, 74.58; H, 9.51; S, 7.96. Found (%): C, 74.60; H, 9.62; S, 8.10. 

3-((Pyridin-2-ylthio)methyl)-4,6-di-*tert*-butylcatechol (**11**). Yield 0.608 g (88%). Beige powder with m.p. 180–182 °C. IR (KBr, ν/cm^−1^): 3458, 3052, 3028, 2992, 2957, 2913, 2869, 1584, 1560, 1455, 1417, 1385, 1356, 1417, 1385, 1356, 1295, 1247, 1225, 1202. ^1^H NMR (400 MHz, DMSO-d_6_, δ, ppm): 1.33 (s, 9 H, tBu), 1.35 (s, 9 H, tBu), 4.61 (s, 2 H, CH_2_), 6.76 (s, 1 H, C_6_H_1_ arom.), 7.16 (ddd, ^3^J(H,H) = 7.3 Hz, ^3^J(H,H) = 5.0 Hz, ^4^J(H,H) = 0.9 Hz, 1H, C_5_H_4_N), 7.34 (dt, ^3^J(H,H) = 8.1 Hz, ^4^J(H,H) = 0.9 Hz, 1H, C_5_H_4_N), 7.67 (td, ^3^J(H,H) = 7.8 Hz, ^4^J(H,H) = 1.8 Hz, 1H, C_5_H_4_N), 7.96 (br.s, 1H, OH), 8.49 (ddd, ^3^J(H,H) = 5.0 Hz, ^4^J(H,H) = 1.8 Hz, ^4^J(H,H) = 0.9 Hz, 1H, C_5_H_4_N), 9.08 (br.s, 1H, OH). ^13^C NMR (100 MHz, DMSO-d_6_, δ, ppm): 29.06, 29.49, 32.05, 34.59, 35.35, 115.01, 119.85, 120.87, 121.89, 134.08, 137.02, 137.83, 142.79, 145.47, 149.12, 159.64. Calcd. for C_20_H_27_NO_2_S (%): C, 69.53; H, 7.88; N, 4.05; S, 9.28. Found (%): C, 69.49; H, 8.08; N, 4.23; S, 9.31.

3-((5-Methoxybenzoxazol-2-ylthio)methyl)-4,6-di-*tert*-butylcatechol (**12**). Yield 0.324 g (39%). Beige powder with m.p. 178–180 °C. IR (KBr, ν/cm^−1^): 3434, 3099, 3008, 2987, 2967, 2876, 1628, 1611, 1588, 1499, 1456, 1431, 1392, 1352, 1306, 1266, 1227, 1188. ^1^H NMR (400 MHz, CDCl_3_, δ, ppm): 1.41 (s, 9 H, tBu), 1.46 (s, 9 H, tBu), 3.88 (s, 3 H, OCH_3_), 4.88 (s, 2 H, CH_2_), 6.49 (s, 1H, C_6_H_1_ arom.), 6.87 (dm, ^3^J(H,H) = 8.8 Hz, 1H, C_6_H_3_ arom.), 6.98 (s, 1H, OH), 7.14 (m, 1H, C_6_H_3_ arom.), 7.32 (dm, ^3^J(H,H) = 8.8 Hz, 1H, C_6_H_3_ arom.), 11.20 (s, 1H, OH). ^13^C NMR (100 MHz, CDCl_3_, δ, ppm): 29.32, 31.55, 32.95, 34.88, 36.24, 56.02, 101.19, 110.43, 112.71, 117.19, 120.65, 134.02, 138.17, 140.43, 143.47, 143.72, 146.61, 157.61, 169.03. Calcd. for C_23_H_29_NO_4_S (%): C, 66.48; H, 7.03; N, 3.37; S, 7.72. Found (%): C, 66.37; H, 7.36; N, 3.32; S, 7.95.

8-(4,6-Di-*tert*-butyl-2,3-dihydroxybenzylthio)quinolinium chloride (**13**). Yield 0.475 g (55 %). Brown powder with m.p. 224–226 °C. IR (KBr, ν/cm^−1^): 3472, 3052, 2995, 2957, 2910, 2869, 2639, 1607, 1563, 1492, 1467, 1417, 1364, 1329, 1289, 1255, 1218. ^1^H NMR (400 MHz, DMSO-d_6_, δ, ppm): 1.37 (s, 9 H, tBu), 1.39 (s, 9 H, tBu), 4.46 (s, 2 H, CH_2_), 6.78 (s, 1H, C_6_H_1_ arom.), 7.58-7.66 (m, 2H, C_9_H_6_N), 7.86 (d, ^3^J(H,H)=7.7 Hz, 2H, C_9_H_6_N), 7.95 (s, 1H, OH), 8.46 (dm, ^3^J(H,H)=8.2 Hz, 1H, C_9_H_6_N), 8.93 (m, 1H, C_9_H_6_N), 9.47 (br.s, 1H, OH). ^13^C NMR (100 MHz, DMSO-d_6_, δ, ppm): 29.47, 31.53, 32.15, 34.57, 35.38, 114.66, 119.37, 122.19, 125.61, 127.03, 128.09, 133.88, 137.45, 137.89, 142.61, 145.00, 146.00, 149.40. Calcd. for C_24_H_30_ClNO_2_S (%): C, 66.72; H, 7.00; Cl, 8.21; N, 3.24; S, 7.42. Found (%): C, 66.82; H, 7.23; Cl, 8.27; N, 3.53; S, 7.40.

3-(Adamantane-1-yl-thio)-4,6-di-*tert*-butylcatechol (**14**). Yield 0.536 g (46%). White powder with m.p. 150–152 °C. IR (KBr, ν/cm^−1^): 3522, 3282, 2961, 2943, 2905, 2848, 1492, 1448,1391, 1366, 1354, 1294, 1264, 1239, 1210. ^1^H NMR (CDCl_3_, 400 MHz, δ, ppm): 1.41 (s, 9H, tBu), 1.48 (s, 9H, tBu), 1.65 (br.t, ^3^J(H,H) = 2.9 Hz, 6H, 3CH_2_), 1.93 (br.t, ^3^J(H,H) = 2.6 Hz, 6H, S-C(CH_2_)_3_), 2.02 (br.m, 3Н, 3CH), 5.53 (s, 1H, OH), 6.92 (s, 1H, C_6_H_1_ arom.), 7.14 (s, 1H, OH). ^13^C{^1^H} NMR (CDCl_3_, 100 MHz, δ, ppm): 29.33, 30.64, 32.46, 35.06, 35.98, 37.00, 44.54, 52.41, 112.91, 116.11, 135.73, 140.08, 144.20, 146.10. HR-MS: Found *m*/*z*: 387.2365 [M + H]^+^. C_24_H_35_O_2_S. Calcd. *m*/*z*: 387.2363.

3-(Benzo[d]thiazol-2-ylthio)-4,6-di-*tert*-butylcatechol (**15**). Yield 0.674 g (58%). White powder with m.p. 190–192 °C. IR (KBr, ν/cm^−1^): 3495, 3083, 3066, 2991, 2954, 2907, 2869, 1560, 1476, 1464, 1427, 1396, 1371, 1313, 1290, 1267, 1238, 1206, 1180, 1156. ^1^H NMR (200 MHz, CDCl_3_, δ, ppm): 1.46 (s, 9H, tBu), 1.49 (s, 9H, tBu), 6.13 (s, 1H, OH), 7.08 (s, 1H, C_6_H_1_ arom.), 7.28 (t, ^3^J(H,H) = 7.6 Hz, 1H, C_6_H_4_ arom.), 7.41 (t, ^3^J(H,H) = 7.6 Hz, 1H, C_6_H_4_ arom.), 7.66 (d, ^3^J(H,H) = 7.8 Hz, 1H, C_6_H_4_ arom.), 7.85 (d, ^3^J(H,H) = 7.8 Hz, 1H, C_6_H_4_ arom.), 8.61 (br.s, 1H, OH). ^13^C{^1^H} NMR (50 MHz, CDCl_3_, δ, ppm): 29.24, 31.53, 35.32, 36.90, 117.43, 121.05, 121.73, 124.82, 126.37, 137.90, 142.71, 143.09, 146.12, 153.00 (some weak signals have not been accumulated). Calcd. for C_21_H_25_NO_2_S_2_ (%): C, 65.08; H, 6.50; N, 3.61; S, 16.54. Found (%): C, 65.10; H, 6.70; N, 3.67; S, 16.49.

### 3.4. Antioxidant Activity Assays

#### 3.4.1. DPPH Radical Scavenging Activity Assay

DPPH radical scavenging activity was performed according to the method of Bondet et al. [84] with some modification. A MeCN solution (C_0_ = 50 µmol) of the radical DPPH was prepared daily and protected from light. For each compound, different concentrations were tested (expressed as the number of moles of antioxidant/mole DPPH). The antioxidant solution in acetonitrile (0.05 mL) was added to 2 mL of a 50 µmol solution of DPPH in MeCN. The decrease in absorbance was determined at 517 nm at 0, 1, 2, 3, 4, 5 min and every 5 min until the reaction reached a plateau at room temperature. 

The parameter EC_50_ is the concentration of an antioxidant necessary for decreasing the amount of DPPH radical by 50% of the initial value. The value of EC_50_ was determined at equilibration in 0–10 min depending on the compound used. To determine EC_50_, the plot of the residual concentration of the stable radical vs. molar ratio, expressed as the number of moles of the antioxidant per 1 mole of the DPPH, was constructed. The parameter (n_DPPH_) is the number of molecules of converted DPPH radical per one molecule of the compound (n_DPPH_ = C_0_/(2 × EC_50_), where C_0_ is the initial concentration of radical). TEC_50_ is the time of achievement of an equilibrium state at the antioxidant concentration equal to EC_50_. The antiradical efficiency (AE) was determined with the equation AE = 1/(EC_50_ × TEC_50_) [85]. To determine EC_50_, the plot of the residual concentration of the stable radical vs. molar ratio, expressed as the number of moles of the antioxidant per 1 mole of the stable radical, was constructed. All experiments were performed in triplicate at room temperature.

#### 3.4.2. ABTS Assay

The radical cation ABTS^∙+^ is generated by the oxidation of ABTS with the K_2_S_2_O_8_. The reduction in the intensity of greenish coloration characteristic of this radical reflects the ability of the antioxidants to scavenge the radical cation [86]. ABTS was dissolved in water to a 7 mM concentration. ABTS radical cation was produced by reacting ABTS stock solution with 2.45 mM potassium persulfate (final concentration) and allowing the mixture to stand in the dark at room temperature for 12–20 h before use. This produced a solution of ABTS^•+^ which gave an absorbance at 734 nm. 1.0 mM stock solutions of test compounds were prepared. Ethanol was the solvent for all compounds with exception of catechol **2** (DMSO). A 7 mM ABTS solution (40 μL) was added to 2.5 mL of ethanol, and the optical density was measured at 734 nm at room temperature. The optical density of the alcohol solution was in the range of 0.70–0.72 (A_0_). Then various aliquots of test substances were added, the final concentration of which varied from 1 to 40 µM. The absorbance (A_i_) reading was taken at the room temperature exactly 1 min after initial mixing and up to 6 min. All measurements were carried out at least three times. Plots of absorbance versus concentration were prepared for test compounds and Trolox. TEAC values were measured by comparing the slopes of plots obtained for each compound compared to that of Trolox. The absorbance of the blank (40 μL ethanol (DMSO) and 40 μL of radical cation) assay was set as 100% radical. The IC_50_ values were calculated as the minimum concentration of each sample required to inhibit 50% of the ABTS radical.

#### 3.4.3. CUPRAC Assay

Cu(II) ion reducing (CUPRAC) assay was carried out in accordance with a known method [87]. The solution of CuCl_2_ (0.01 M) is prepared by dissolving in aqueous ammonium acetate buffer (1 M). Neo-cuproine (Nc) (7.5 mM) is dissolved in 96% ethanol. In a test tube 0.5 mL each of Cu(II), Nc, and ammonium acetate buffer solutions were added. The catechol thioether or Trolox solution (ethanol or DMSO) and ethanol (96%) were added to the initial mixture so as to make the final volume 2 mL. The concentration of compounds in test tubes ranged from 10 to 50 μM. Absorbance was measured at 450 nm on spectrophotometer Akvilon SF-104 (Russia) against a reagent blank 30 min later. The standard calibration curves of each antioxidant compound were constructed by plotting absorbance versus molar concentration. Trolox was used as the standard antioxidant for calculate TEAC (Trolox Equivalent Antioxidant Capacity). TEAC coefficient for this assay was determined by relating the molar absorptivity, ε of the test samples to that of Trolox as follows: TEAC = ε/ε_Trolox_ (ε_Trolox_ = 1.79·10^4^ L·mol^−1^·cm^−1^).

#### 3.4.4. Inhibition of Superoxide Radical Anion Formation by Xanthine Oxidase (NBT Assay)

To evaluate the radical scavenging activity of compounds, a reaction catechol thio-ether with O_2_^∙−^ generated in the xanthine/xanthiooxidase enzymatic system was used. This reaction is based on the ability of tetrazolium blue to be reduced to formazan upon interaction with the superoxide anion radical. The reaction mixture consisted of 2.70 mL of 40 mM sodium carbonate buffer containing 0.1 mM EDTA (pH 10.0), 0.06 mL of 10 mM xanthine, 0.03 mL of 0.5% bovine serum albumin, 0.03 mL of 2.5 mM nitroblue tetrazolium and 0.06 mL of the sample solution in ethanol or DMSO. To the mixture at 22 °C 0.05 mL of xanthine oxidase (0.04 units) was added, and the absorbance at 560 nm (by formation of blue formazan) [88] was recorded by Thermo Scientific Multiskan Sky microplate spectrophotometer for 800 s. A control experiment was carried out by replacing the sample solution with the same amount of ethanol or DMSO. Inhibition I (%) = ((1 − A_i_/A_0_) × 100%), where A_i_ is the absorbance in the presence of catechol thioether at the end of the reaction (800 s), A_0_ is the absorbance of the blank solution. The IC_50_ values were determined graphically using the dependence of inhibition values versus the concentration of the compound. All experiments were performed three times.

#### 3.4.5. AAPH-Induced Oxidation of DNA Assay

AAPH-induced oxidation of DNA (the deoxyribonucleic acid sodium salt from salmon testes) was carried out following the known method with a little modification [89]. Briefly, 0.02 mL of stock solutions of the research compounds in DMSO were added to PBS (pH 7.4) solutions of AAPH and DNA, in which the final concentration of DNA and AAPH was kept 2.5 mg∙mL^−1^ and 40 mmol, respectively. Then, the above solution was dispatched into test tubes with 2.0 mL solution contained in each one. All the tubes were incubated in a water bath during 2.5 h at 37 °C to initiate the oxidation. Test tubes were taken out and cooled immediately, to which 1.0 mL of TBA (1.00 g TBA and 0.40 g NaOH dissolved in 100 mL PBS (pH 7.4)) and 1.0 mL of 3.0% trichloroacetic acid aqueous solution was added. The tubes were heated in a boiling water bath for 15 min. After cooling, 2.0 mL of *n*-butanol was added and shaken vigorously to extract TBARS. The absorbance of n-butanol layer was measured at 535 nm. Finally, the average value of three absorbance data (within 10% experimental error) was determined. The absorbance in the blank experiment and in the presence of the compounds **1**–**15**, or Trolox were assigned as A_0_ and A_i_. The antioxidant effect of the tested compounds (in percentage of forming TBARS) on the AAPH induced oxidation of DNA was expressed by (1 − A_i_/A_0_) × 100.

#### 3.4.6. Lipid Peroxidation of Rat Liver Homogenate

Samples of rat Wistar liver were homogenized (1:10 *w*/*v*) in phosphate buffer, pH 7.4 using a homogenizer. The extent of lipid peroxidation was estimated by using the thio-barbituric acid reactive substances (TBARS) assay [77]. The influence of compounds **1**–**10**, **12**, **13** on lipid peroxidation of the rat liver homogenates was carried out at 37 °C for 3 h in phosphate buffer (pH 7.4) in the presence or absence of compounds or vehicle (CHCl_3_). The concentration of compounds in the medium was 0.1 mM. The level of lipid peroxidation was measured in the rat (Wistar) liver homogenates as a non-enzymatic process by the addition of ascorbic acid and (NH_4_)_2_Fe(SO_4_)_2_. The homogenate was divided into following experimental groups: one control homogenate and five samples of homogenate with the addition of target compounds. Solutions of ascorbic acid (0.1 mL, 2.6 mmol), Fe(NH_4_)_2_(SO_4_)_2_ (0.1 mL, 0.4 mM) and trichloroacetic acid (1 mL, 40%) were injected into the probe. The test tubes were incubated at 37 °C and then probes were centrifuged (10 min at 3000× *g*). The supernatants (2 mL) were transferred to new test tubes and mixed with 1ml TBA solution (0.8 %). The probes were heated at 95 °C for 10 min, cooled at 4 °C, then probes centrifuged (10 min at 10,000× *g*) and the supernatants absorbance was measured at 535 nm at Thermo Scientific Multiskan Sky microplate spectrophotometer. All the experiments were performed using three independent experiments. Data are normalized to control probe with oxidant. Preliminary experiments were done in the absence of compounds interaction with thio-barbituric acid. The values are expressed as mean % ± SD.

## 4. Conclusions

Thus, we have synthesized a series of new catechol thio-ethers containing a methylene linker between the catechol ring and the thioether group. The reaction of sterically hindered methoxy-catechol with thiols occurs in acetic acid with the formation of target compounds. The yields reach up to 83%. The molecular structure of thio-ethers with a benzo-thiazole group in crystalline state was established by single-crystal X-ray diffraction analysis. We have found that the introduction of the methylene group affects the intra- or intermolecular hydrogen bonds in the formed dimers. Generally, the synthesized compounds are characterized by heterocyclic fragments that belong to biologically active scaffolds and have a wide range of pharmacological activity. The compounds’ radical scavenging and antioxidant activities were evaluated in tests with synthetic radicals, superoxide anion radical, CUPRAC assay, in the lipid peroxidation process and promoted damage of DNA molecules (in vitro). An unambiguous effect of the methylene linker presence on the antioxidant properties for pairs of compounds **10** and **14**, **8** and **15** was not established. Compound **14** is a more active radical scavenger than **10** in the DPPH, ABTS tests, in the process of oxidative damage of DNA molecules, but it has a pronounced promoting effect on the LP reaction. At the same time, **8** is a more active inhibitor of DNA molecules damage than **15**, and it exhibits an antioxidant effect in the LP process. The analysis of antiradical and antioxidant properties made it possible to identify the most promising catechol thio-ethers with thiazole, thiazoline, benzo-thiazole, or benzoxazole groups, which have high activity in all the tests under consideration. Such behavior of these substances allows us to consider them as potential cyto-protectors.

## Figures and Tables

**Table 1:** The values of EC_50_, TEC_50_, AE (antiradical efficiency) in DPPH test (MeCN, 298 K), index of IC_50_ and TEAC in ABTS^∙+^ assay, Cu^2+^ reduced activity in Trolox equivalents (CUPRAC) and superoxide radical anion scavenging activity of target compounds in IC_50_ values.

Compounds	DPPH			ABTS^∙+^		CUPRAC	O_2_^−∙^
	EC_50_, µmol	TEC_50_, min	AE∙10^3^	IC_50_, µmol	TEAC	TEAC	IC_50_, µmol
**1**	13.0 ± 0.9	10.0	7.5 ± 0.5	12.0 ± 0.7	1.03 ± 0.09	1.66 ± 0.10	>100.0
**2**	7.5 ± 0.3	5.0	26.1 ± 2.6	* 15.1 ± 0.9	* 1.49 ± 0.20	* 0.72 ± 0.04	>100.0
**3**	13.0 ± 1.2	30.0	2.5 ± 0.2	12.0 ± 0.8	2.26 ± 0.15	1.10 ± 0.10	n/a **
**4**	21.5 ± 1.5	<1.0	>46.5	21.6 ± 1.8	1.17 ± 0.08	0.83 ± 0.06	34.0 ± 0.1
**5**	12.4 ± 0.7	15.0	5.4 ± 0.3	16.5 ± 0.5	1.27 ± 0.06	0.96 ± 0.07	37.6 ± 0.2
6	17.5 ± 0.6	20.0	2.7 ± 0.1	20.0 ± 1.0	1.05 ± 0.07	0.98 ± 0.05	92.1 ± 0.3
**7**	23.2 ± 1.7	21.0	2.1 ± 0.2	16.0 ± 0.6	1.65 ± 0.04	1.24 ± 0.08	48.2 ± 0.1
**8**	20.1 ± 0.6	10.0	4.9 ± 0.1	7.5 ± 0.5	2.77 ± 0.19	0.94 ± 0.14	97.9 ± 0.4
**9**	21.2 ± 0.9	5.0	9.4 ± 0.4	9.8 ± 0.9	1.93 ± 0.07	0.88 ± 0.07	>100.0
**10**	19.5 ± 0.5	60.0	0.85 ± 0.1	33.2 ± 0.6	0.95 ± 0.07	0.84 ± 0.05	n/a
**11**	11.5 ± 0.4	23.0	3.7 ± 0.3	17.6 ± 2.0	1.31 ± 0.10	1.25 ± 0.11	>100.0
**12**	8.9 ± 0.1	22.0	5.10 ± 0.4	7.0 ± 0.4	2.93 ± 0.09	2.40 ± 0.15	49.0 ± 0.4
**13**	16.5 ± 0.8	40.0	1.51 ± 0.1	14.1 ± 0.5	1.63 ± 0.04	1.55 ± 0.10	>100.0
**14**	11.7 ± 0.4	40.0	2.14 ± 0.1	12.5 ± 0.5	1.36 ± 0.04	0.97 ± 0.09	n/a
**15**	30.2 ± 1.3	5.0	6.80 ± 1.0	22.6 ± 1.7	1.03 ± 0.11	0.71 ± 0.04	45.8 ± 0.3
Trolox	12.0 ± 0.5	10.3	5.1 ± 0.5	16.0 ± 1.0	1.00 ± 0.03	1.00 ± 0.08	62.7 ± 0.6

* Data obtained in DMSO. ** n/a—non-active.

**Table 2:** Calculated dissociation energies of O–H (D_O–H_) bonds for catechols and ΔH of the reaction with DPPH radical (DFT, basis B3LYP/6-31++G (d, p)).

Compounds	D_O__–__H (I)_, kcal∙mol^−1^	D_O__–__H (II)_, kcal∙mol^−1^	ΔH_(I)_, kcal∙mol^−1^	ΔH_(II)_, kcal∙mol^−1^
**1**	74.35	73.87	−7.35	−7.83
**2**	78.03	84.30	−3.22	3.03
**3**	78.50	88.20	−2.76	6.92
**4**	76.02	83.18	−5.68	1.47
**5**	74.65	74.07	−7.05	−7.63
**6**	77.27	85.10	−4.43	3.39
**7**	77.86	84.93	−3.84	3.22
**8**	84.23	74.66	2.52	−7.04
**9**	77.61	84.67	−4.09	2.96
**10**	75.42	83.59	−5.84	2.33
**11**	74.90	74.26	−6.80	−7.44
**12**	84.24	74.71	2.53	−6.99
**13**	81.32	74.02	−0.39	−7.68
**14**	78.92	79.80	−2.78	−1.90
**15**	84.44	75.64	3.73	−6.06

## Data Availability

The data presented in this study are available in this article.

## Supplementary Materials

The following supporting information can be downloaded at: https://www.mdpi.com/article/10.3390/molecules27103169/s1. Table S1: Crystal data and structure refinement for **8**, and **15**; Figures S1–S28: ^1^H and ^13^C{^1^H} NMR spectra CCDC-2166872 (**8**) and 2166873 (**15**) contain the supplementary crystallographic data for this paper. These data can be obtained free of charge from The Cambridge Crystallographic Data Centre via www.ccdc.cam.ac.uk/data_request/cif (accessed on 13 May 2022).

## Sample Availability

Samples of the compounds **1**–**15** are available from the authors.
