# Supplementary material for: Synthesis and Antioxidant Activity of New Catechol Thioethers with the Methylene Linker
Source: Molecules. 2022 May 16;27(10):3169. doi: 10.3390/molecules27103169 (PMC9144179; doi:10.3390/molecules27103169)
Supplement: Supplementary file 1 [file molecules-27-03169-s001.zip › molecules-1725220-supplementary.pdf]

Supplementary materials

Synthesis and antioxidant activity of new catechol thioethers with the methylene linker

Ivan V. Smolyaninov[*,1], Daria A. Burmistrova[1], Maxim V. Arsenyev[2], Maria A. Polovinkina[3], Nadezhda P. Pomortseva[1], Georgy K. Fukin[2], Andrey I. Poddel'sky[*,2], Nadezhda T. Berberova[1]

[1] Department of Chemistry, Astrakhan State Technical University, 16 Tatisheva str., Astrakhan, 414056, Russia

[2] G.A. Razuvaev Institute of Organometallic Chemistry, Russian Academy of Sciences, 49 Tropinina str., 603137, Nizhny Novgorod, Russia

[3] Toxicology research group of Southern Scientific Centre of Russian Academy of Science, 41 Chekhova str., Rostov-on-Don, 344006, Russia

Content:

Figure S1. The ^{1}H NMR spectrum of **1** (200 MHz, DMSO-d$_6$) . . . S3

Figure S2. The ^{13}C{^{1}H} NMR spectrum of **1** (50 MHz, DMSO-d$_6$) . . . S3

Figure S3. The ^{1}H NMR spectrum of **2** (200 MHz, CDCl$_3$) S4

Figure S4. The ^{13}C{^{1}H} NMR spectrum of **2** (50 MHz, CDCl$_3$) . . . S4

Figure S5. The ^{1}H NMR spectrum of **4** (200 MHz, CDCl$_3$) S5

Figure S6. The ^{13}C{^{1}H} NMR spectrum of **4** (50 MHz, CDCl$_3$) . . . S5

Figure S7. The ^{1}H NMR spectrum of **5** (400 MHz, CDCl$_3$) S6

Figure S8. The ^{13}C{^{1}H} NMR spectrum of **5** (100 MHz, CDCl$_3$) . . . S6

Figure S9. The ^{1}H NMR spectrum of **6** (200 MHz, DMSO-d$_6$) . . . S7

Figure S10. The ^{13}C{^{1}H} NMR spectrum of **6** (50 MHz, DMSO-d$_6$) . . . S7

Figure S11. The ^{1}H NMR spectrum of **7** (200 MHz, CDCl$_3$) S8

Figure S12. The ^{13}C{^{1}H} NMR spectrum of **7** (50 MHz, CDCl$_3$) . . . S8

Figure S13. The ^{1}H NMR spectrum of **8** (200 MHz, CDCl$_3$) S9

Figure S14. The $^{13}C\{^1H\}$ NMR spectrum of **8** (50 MHz, $CDCl_3$) . . . S9

Figure S15. The ^1H NMR spectrum of **9** (400 MHz, $CDCl_3$) S10

Figure S16. The $^{13}C\{^1H\}$ NMR spectrum of **9** (100 MHz, $CDCl_3$) . . . S10

Figure S17. The ^1H NMR spectrum of **10** (200 MHz, $CDCl_3$) S11

Figure S18. The $^{13}C\{^1H\}$ NMR spectrum of **10** (50 MHz, $CDCl_3$) . . . S11

Figure S19. The ^1H NMR spectrum of **11** (400 MHz, DMSO-d_6). . . . S12

Figure S20. The $^{13}C\{^1H\}$ NMR spectrum of **11** (100 MHz, DMSO-d_6). . . S12

Figure S21. The ^1H NMR spectrum of **12** (400 MHz, $CDCl_3$) S13

Figure S22. The $^{13}C\{^1H\}$ NMR spectrum of **12** (100 MHz, $CDCl_3$) . . . S13

Figure S23. The ^1H NM R spectrum of **13** (400 MHz, DMSO-d_6) . . . S14

Figure S24. The $^{13}C\{^1H\}$ NMR spectrum of **13** (100 MHz, DMSO-d_6) . . S14

Figure S25. The ^1H NMR spectrum of **14** (400 MHz, $CDCl_3$). . . . S15

Figure S26. The $^{13}C\{^1H\}$ NMR spectrum of **14** (100 MHz, $CDCl_3$). . . . S15

Figure S27. The ^1H NMR spectrum of **15** (400 MHz, $CDCl_3$). . . . S16

Figure S28. The $^{13}C\{^1H\}$ NMR spectrum of **15** (100 MHz, $CDCl_3$). . . . S16

Table S1. Crystal data and structure refinement for **8** and **15** . . . S17

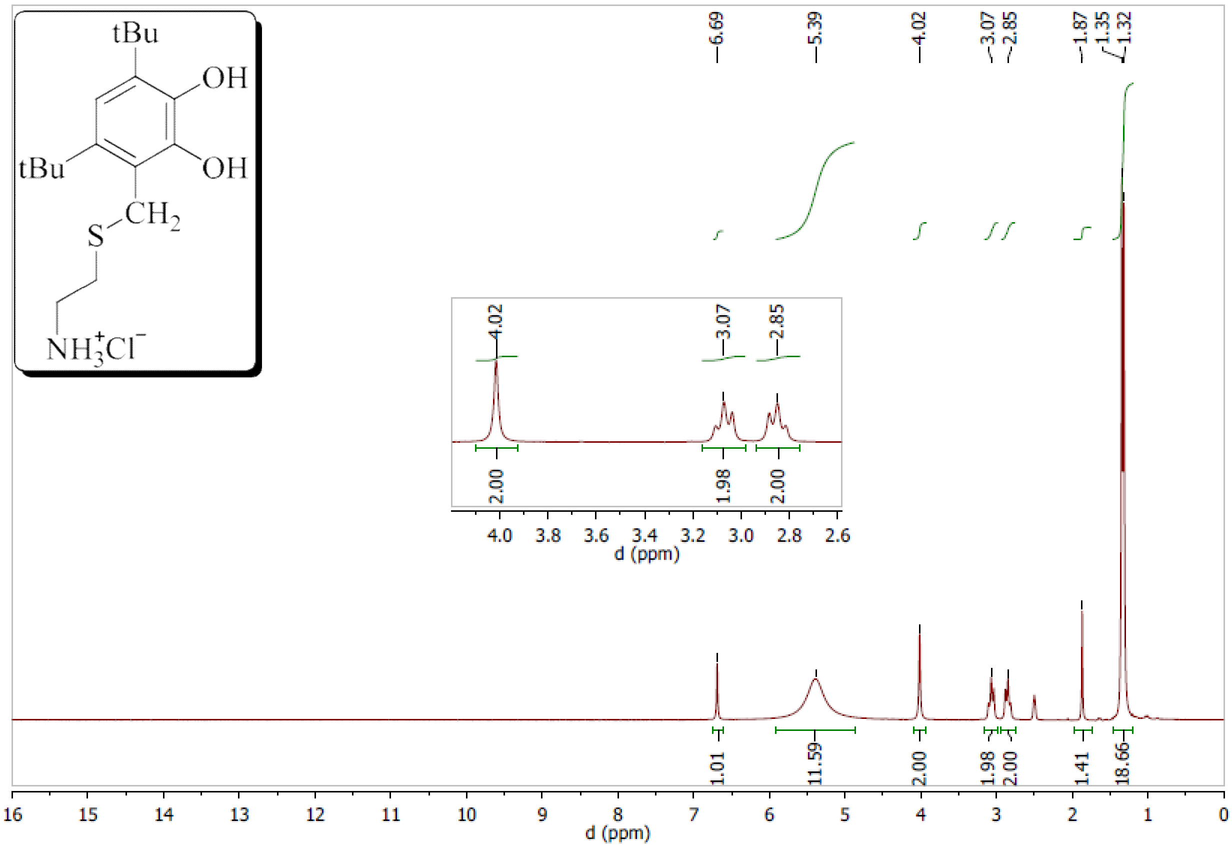

Figure S1. The ^{1}H NMR spectrum of **1** (200 MHz, DMSO-d$_6$).

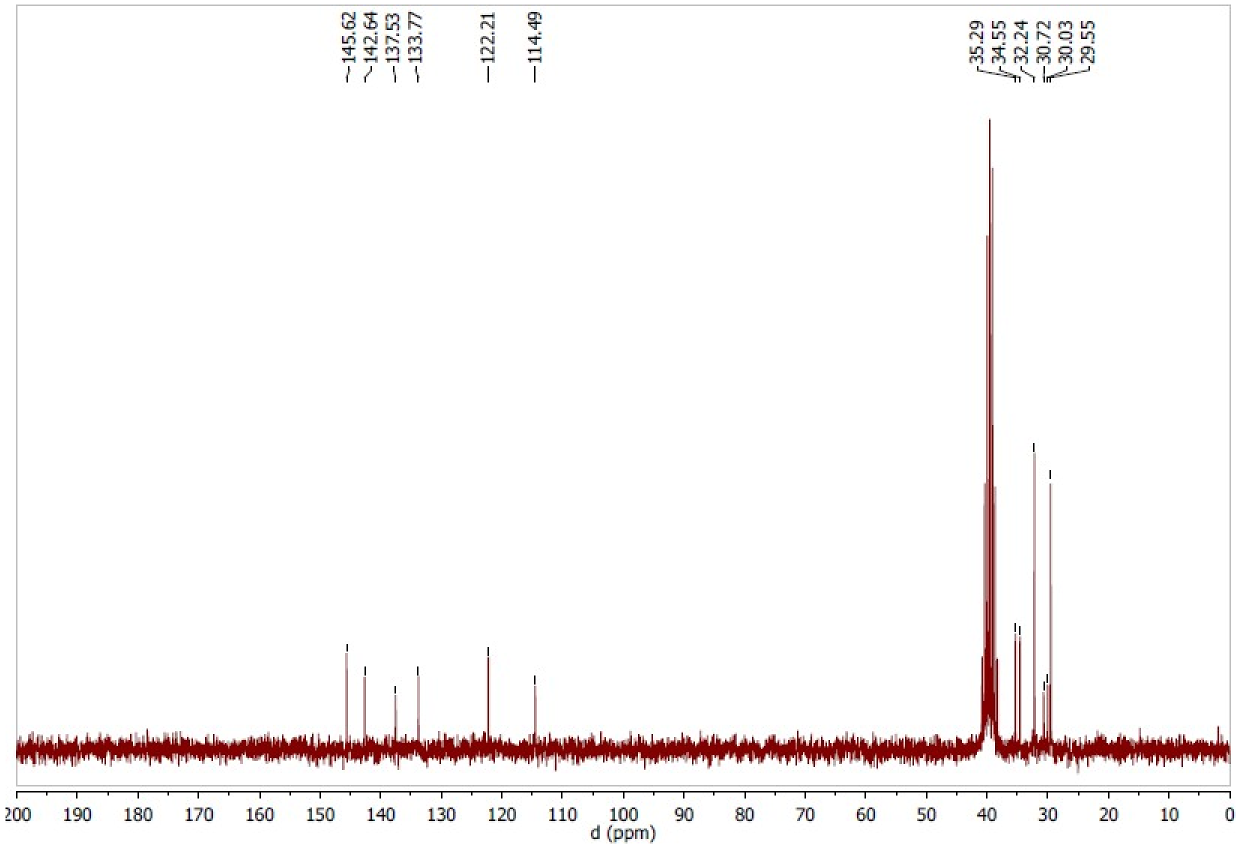

Figure S2. The $^{13}C\{^{1}H\}$ NMR spectrum of **1** (50 MHz, DMSO-d$_6$).

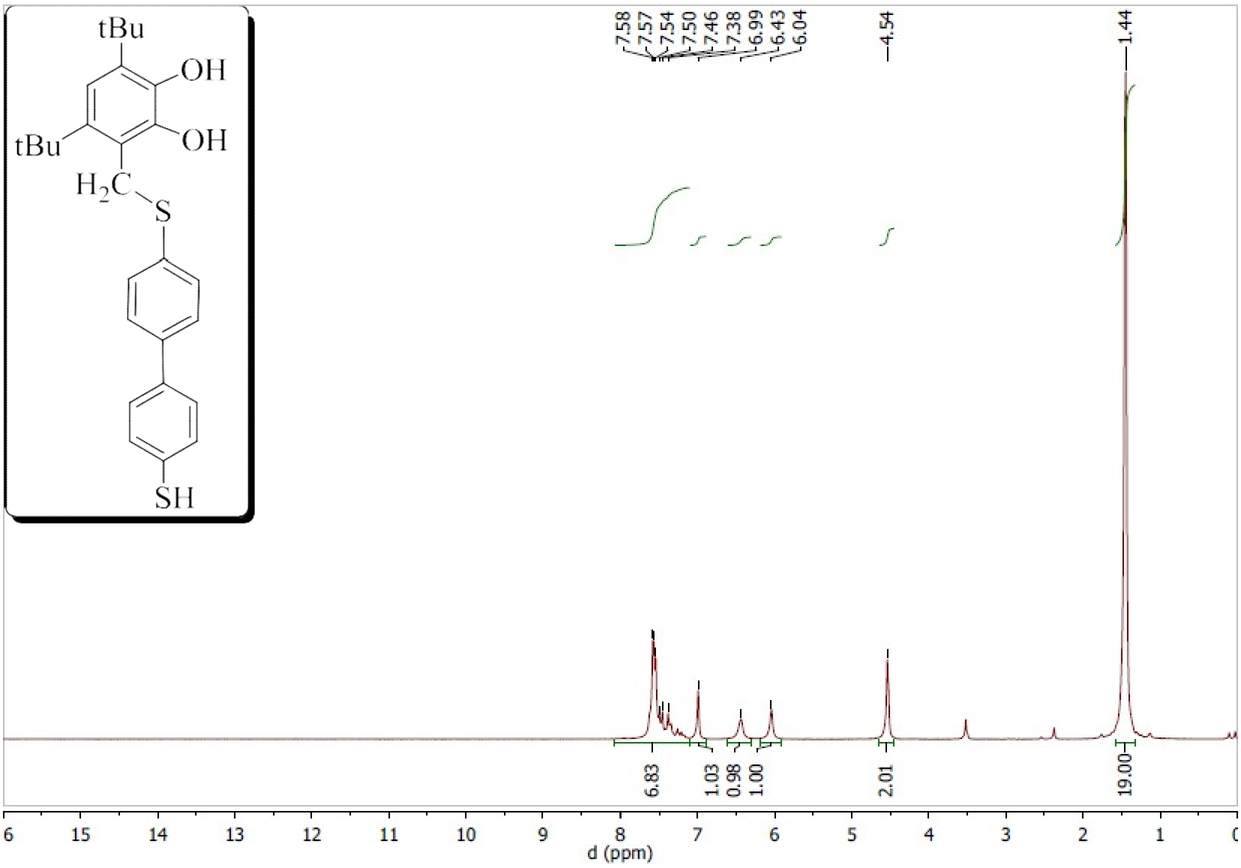

Figure S3. The ^{1}H NMR spectrum of **2** (200 MHz, $CDCl_3$).

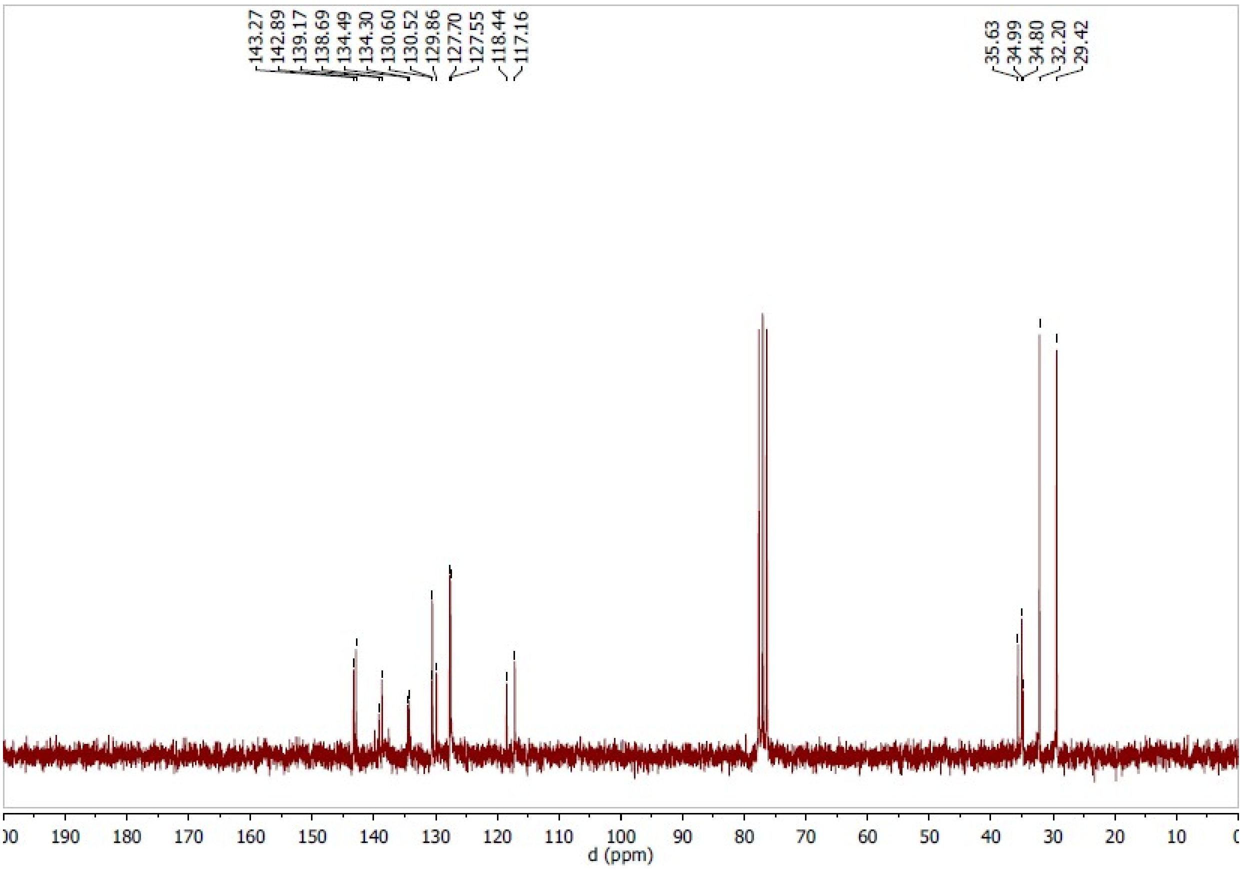

Figure S4. The $^{13}C\{^1H\}$ NMR spectrum of **2** (50 MHz, $CDCl_3$).

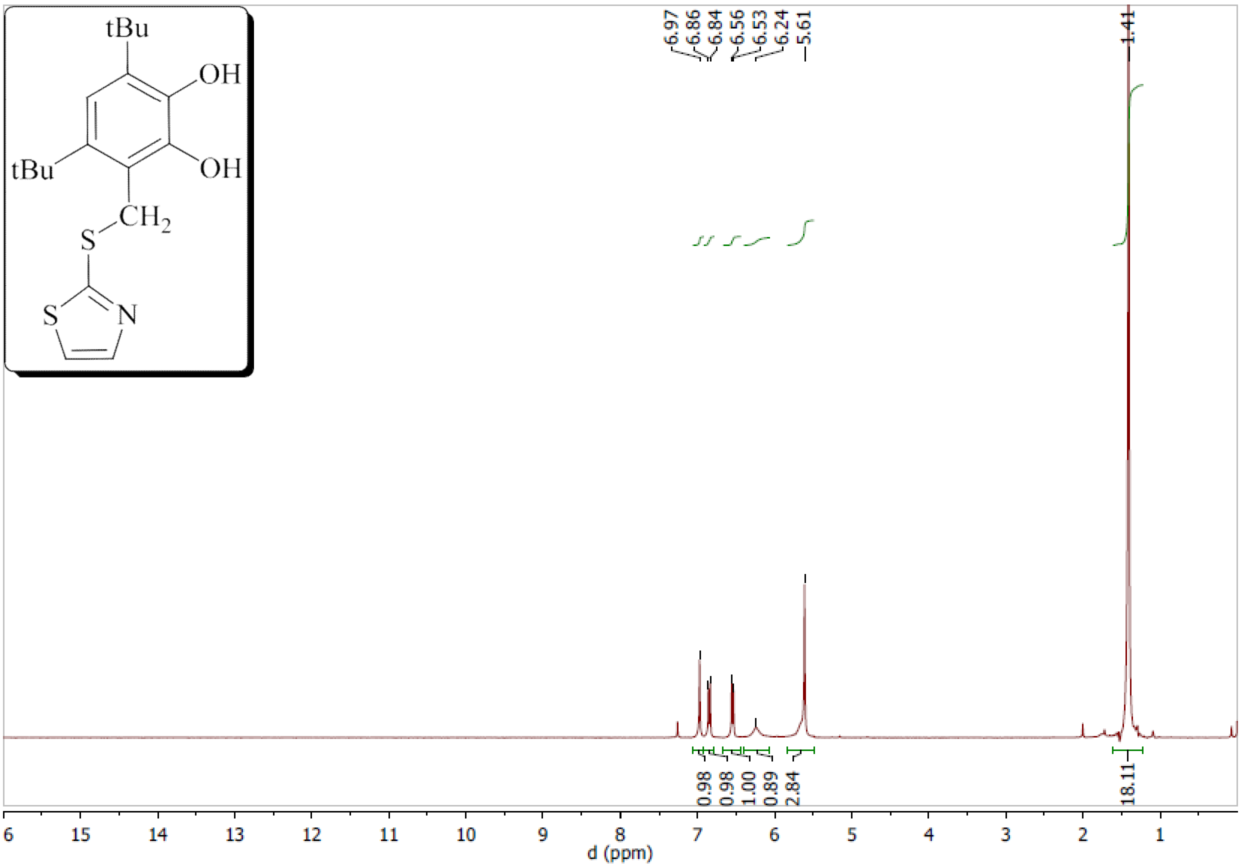

Figure S5. The ^{1}H NMR spectrum of **4** (200 MHz, $CDCl_3$).

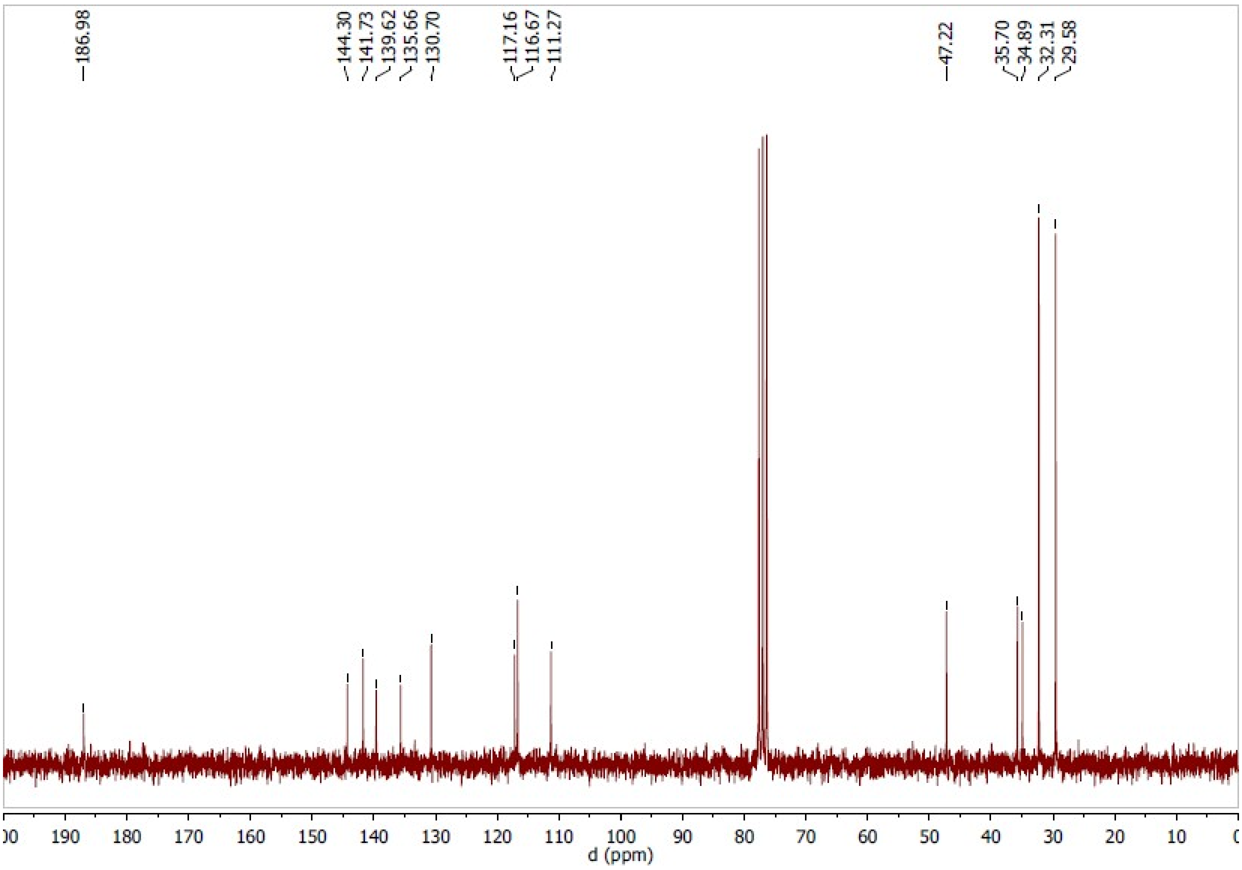

Figure S6. The ^{13}C{^{1}H} NMR spectrum of **4** (50 MHz, $CDCl_3$).

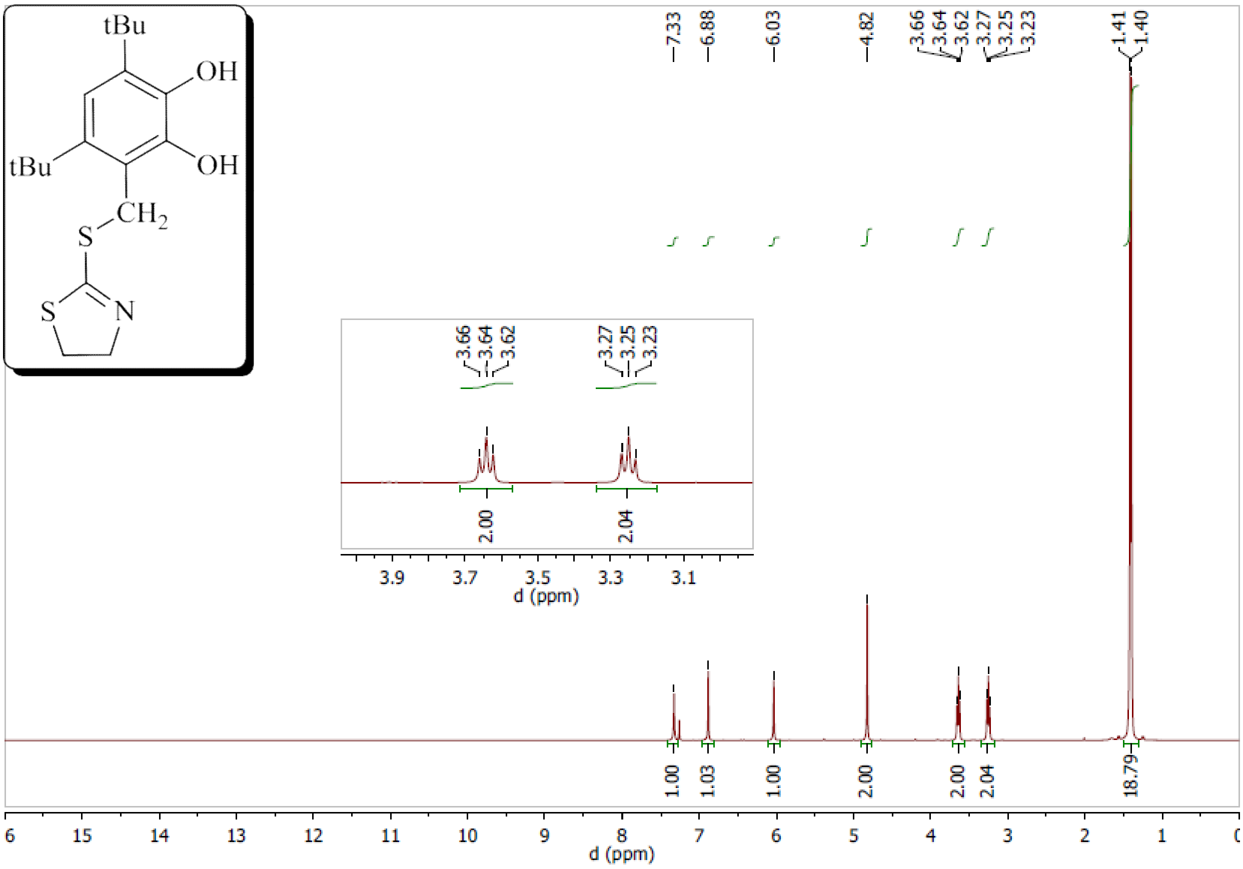

Figure S7. The ^{1}H NM R spectrum of **5** (400 MHz, $CDCl_3$).

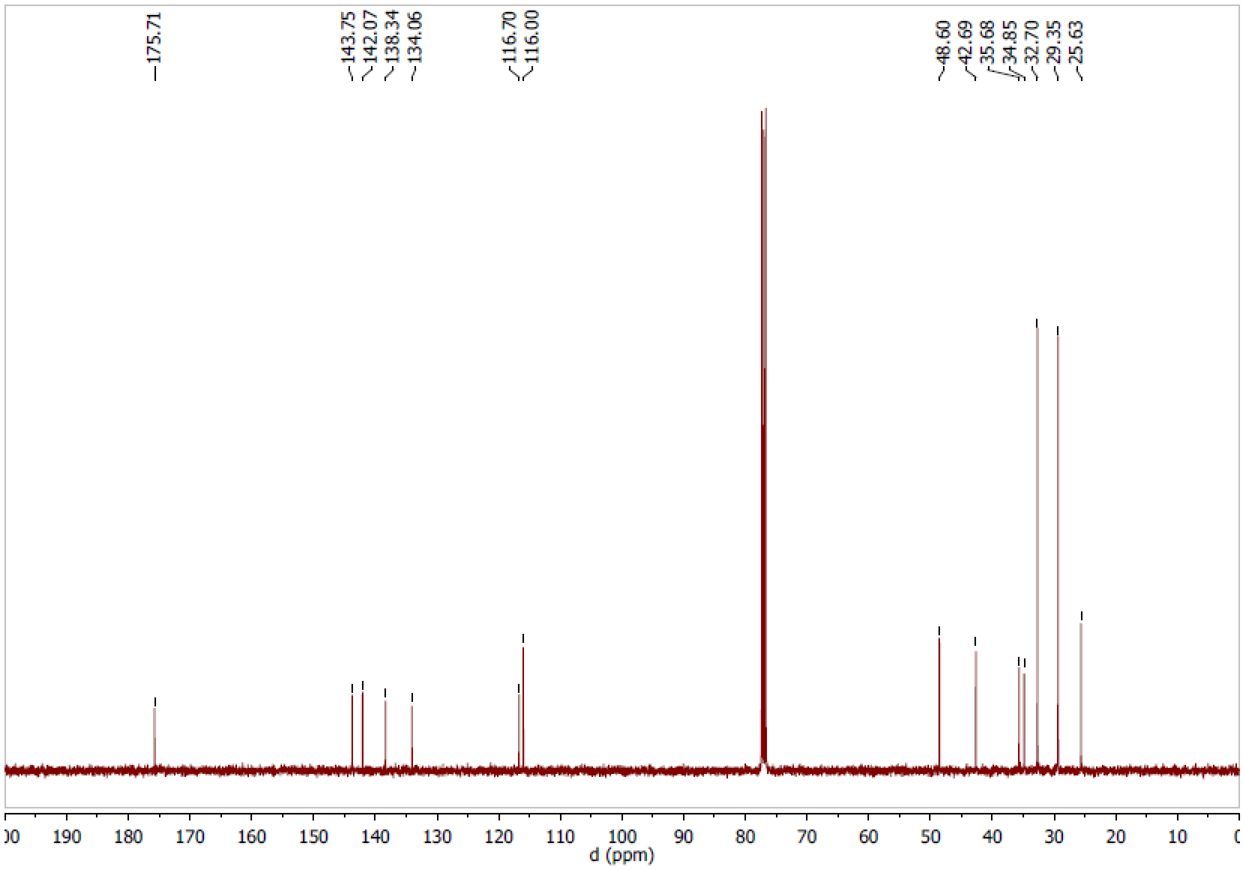

Figure S8. The $^{13}C\{^{1}H\}$ NMR spectrum of **5** (100 MHz, $CDCl_3$).

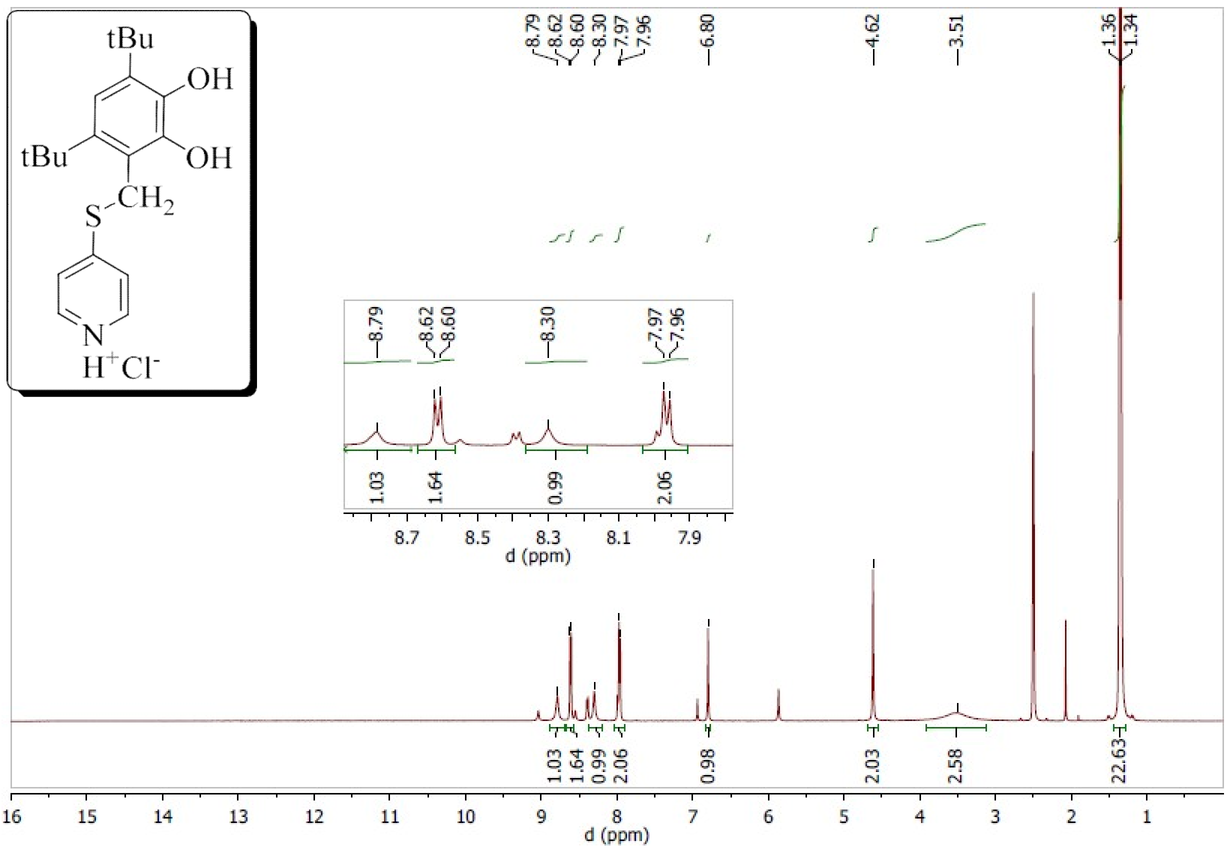

Figure S9. The ^{1}H NMR spectrum of **6** (200 MHz, DMSO-d_6).

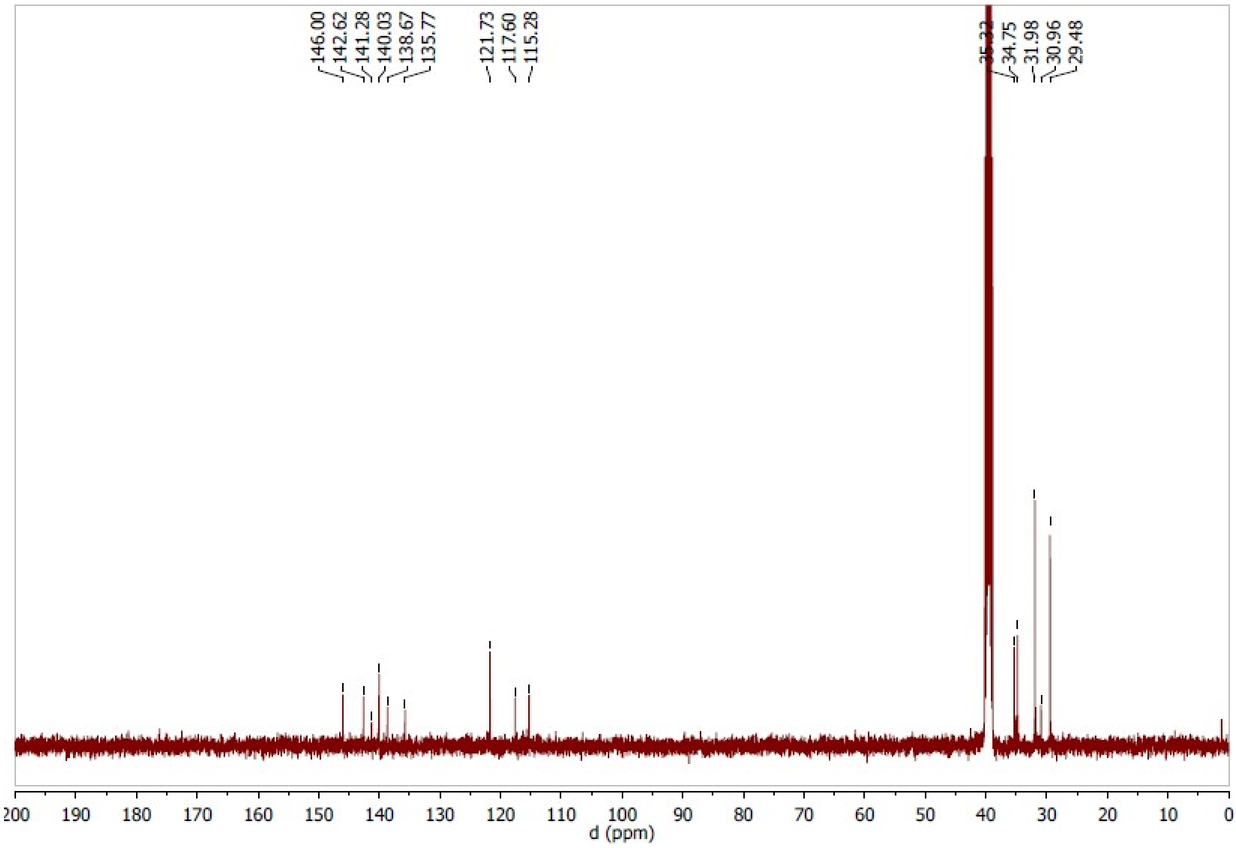

Figure S10. The $^{13}C\{^{1}H\}$ NMR spectrum of **6** (50 MHz, DMSO-d_6).

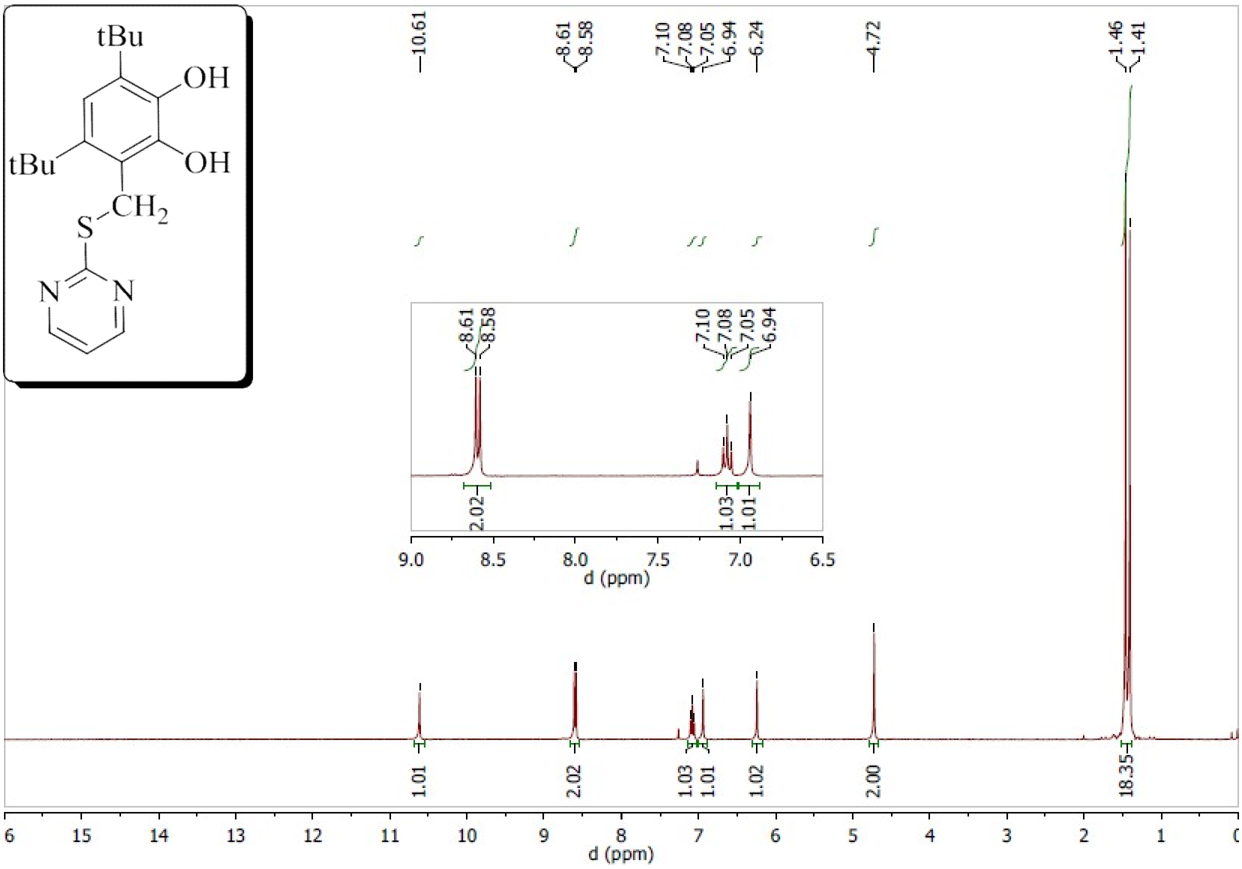

Figure S11. The ^{1}H NMR spectrum of **7** (200 MHz, $CDCl_3$).

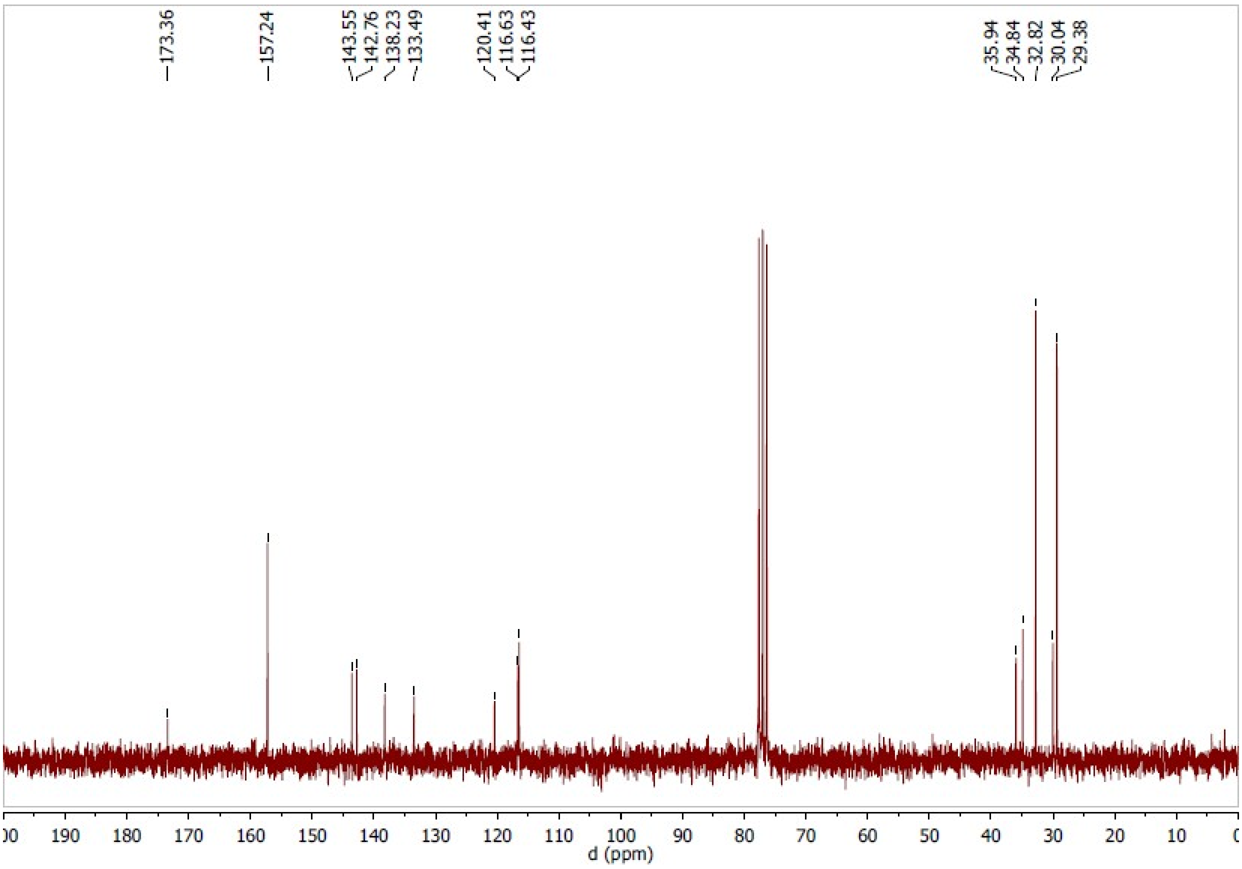

Figure S12. The $^{13}C\{^1H\}$ NMR spectrum of **7** (50 MHz, $CDCl_3$).

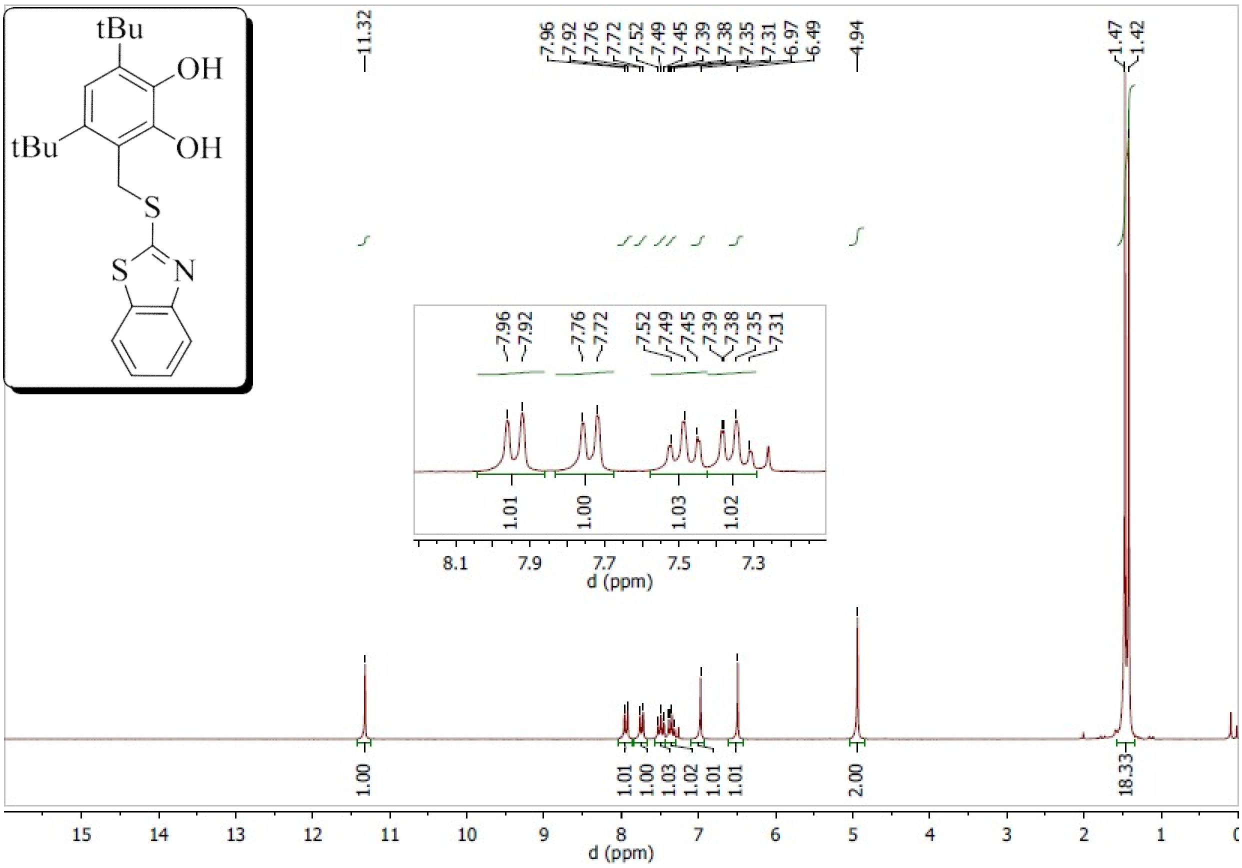

Figure S13. The ^{1}H NMR spectrum of **8** (200 MHz, $CDCl_3$).

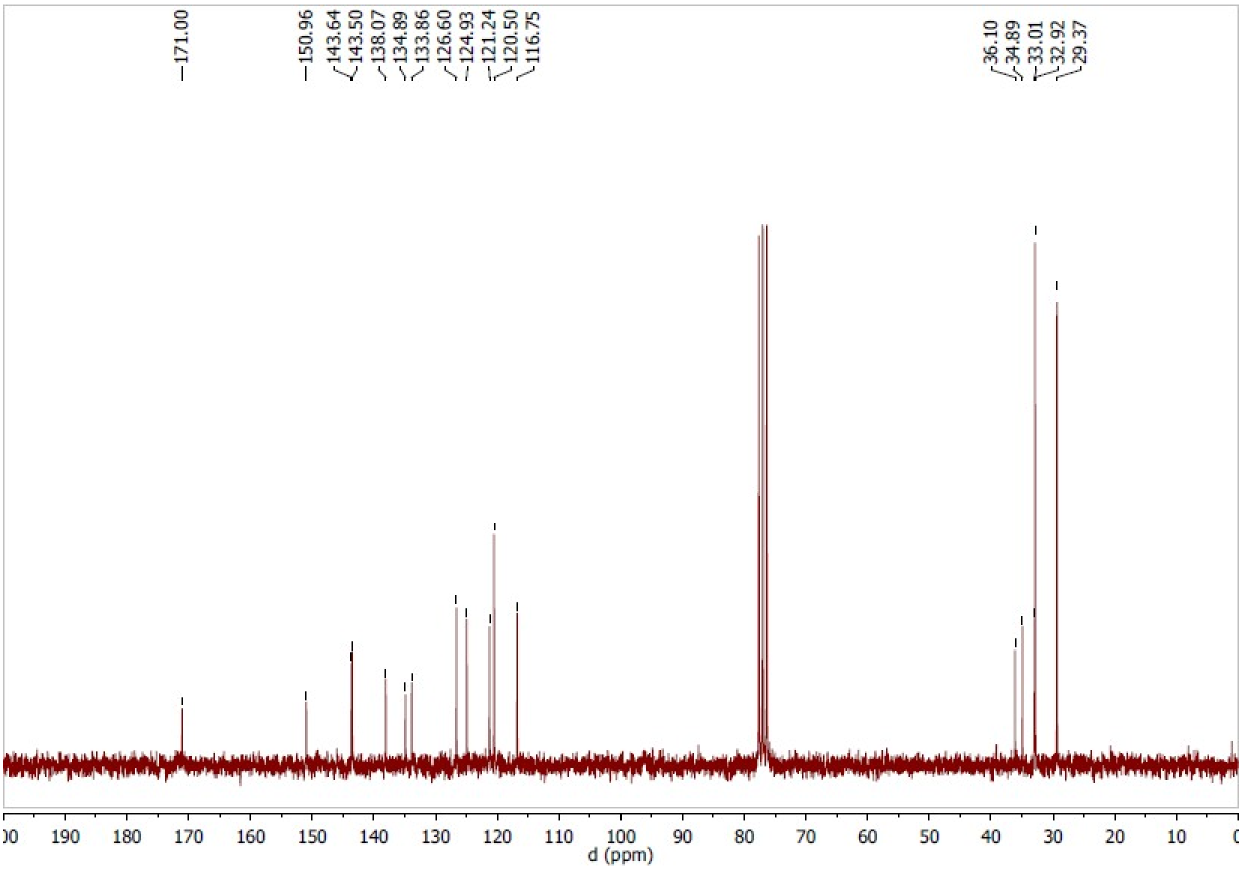

Figure S14. The $^{13}C\{^{1}H\}$ NMR spectrum of **8** (50 MHz, $CDCl_3$).

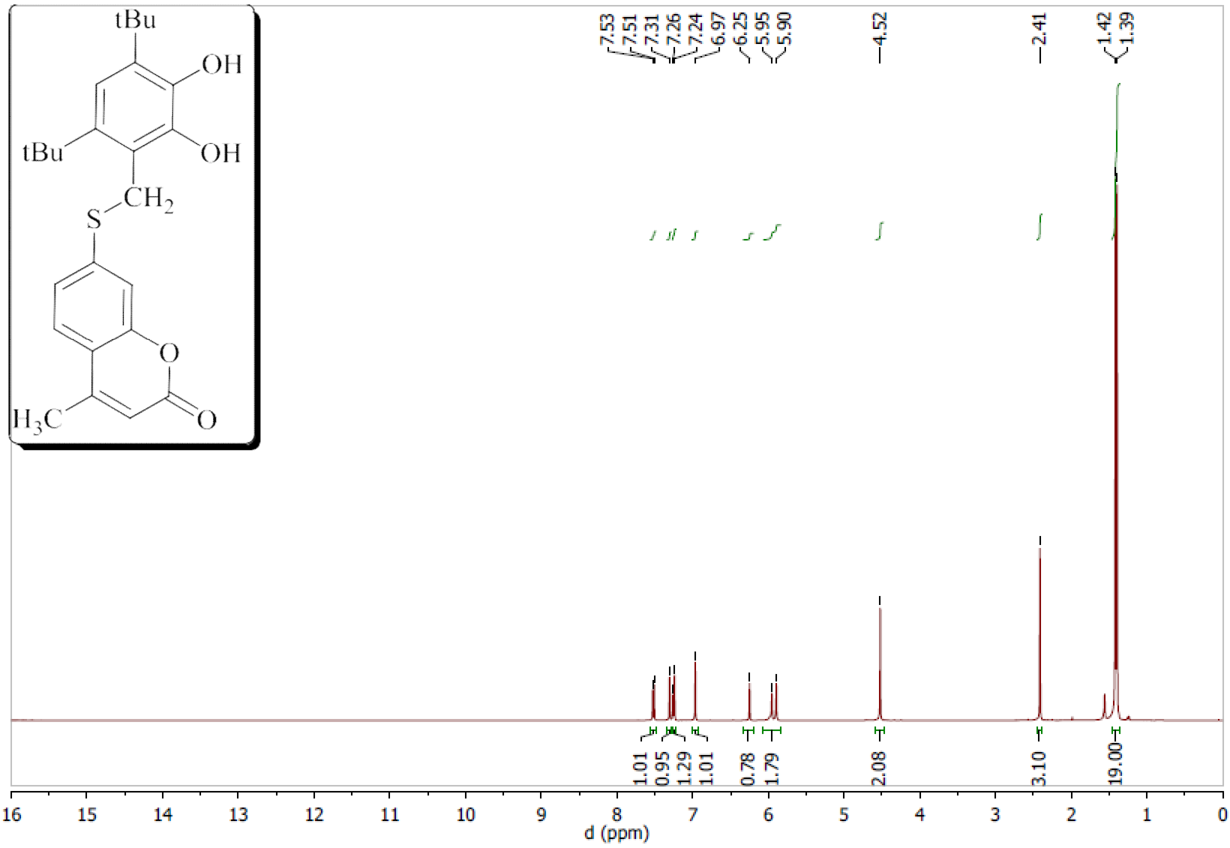

Figure S15. The ^{1}H NMR spectrum of **9** (400 MHz, $CDCl_3$).

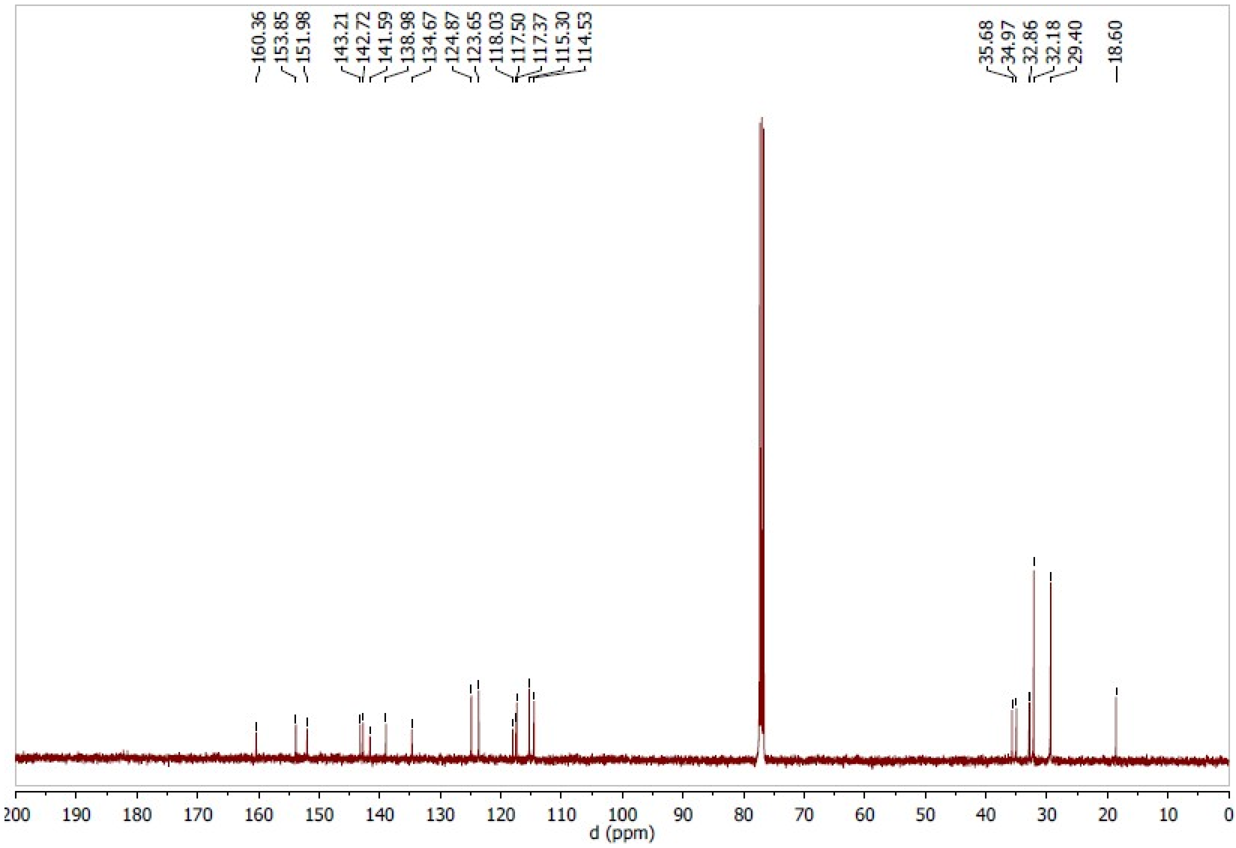

Figure S16. The ^{13}C{^{1}H} NMR spectrum of **9** (100 MHz, $CDCl_3$).

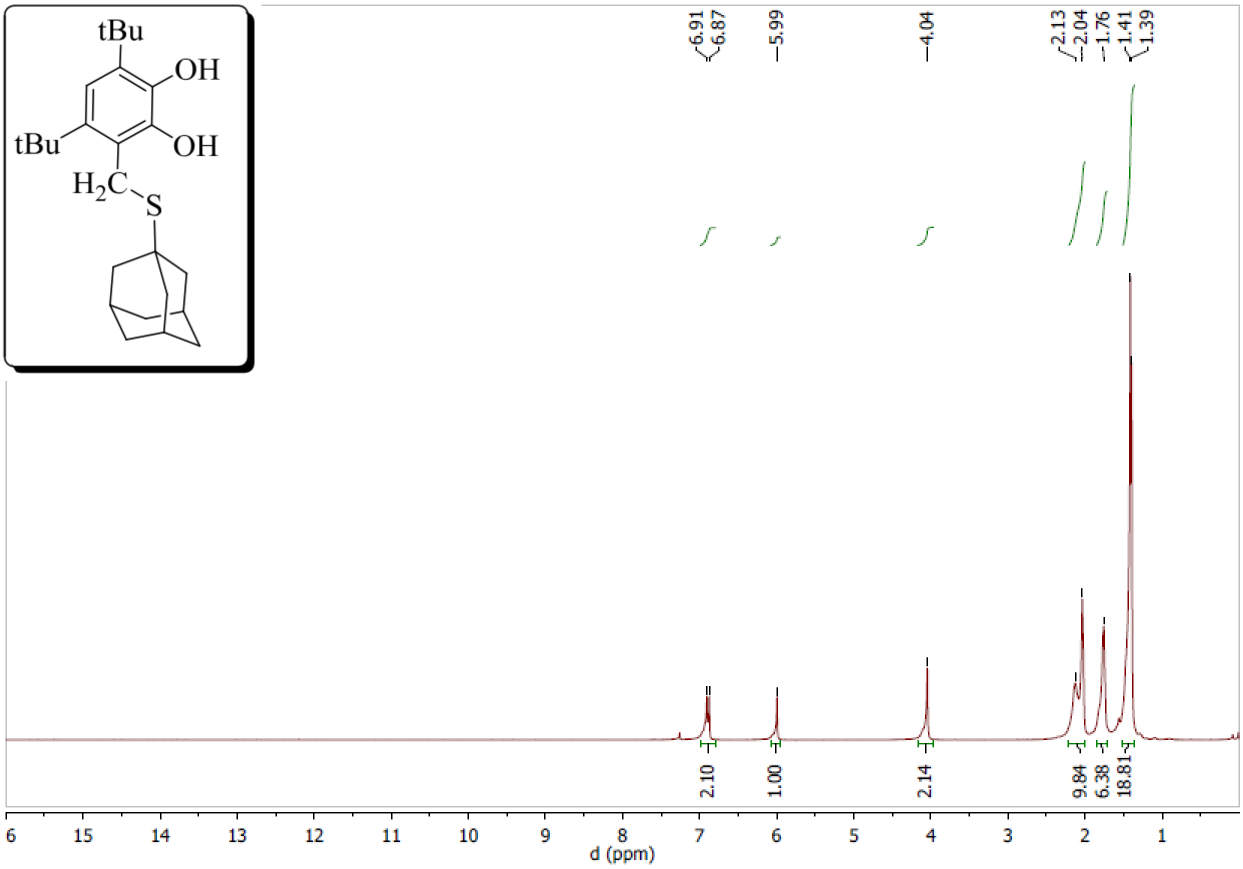

Figure S17. The ^{1}H NMR spectrum of **10** (200 MHz, $CDCl_3$).

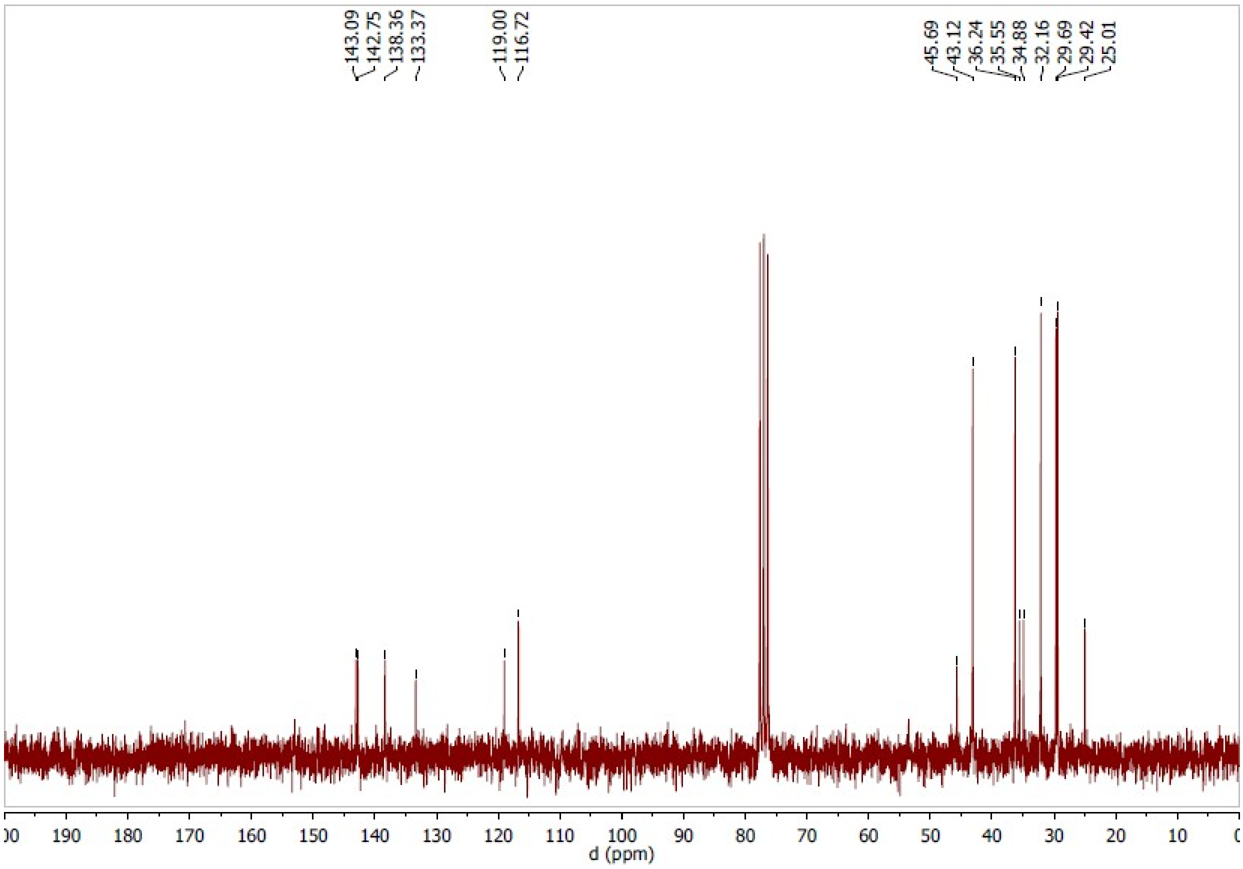

Figure S18. The $^{13}C\{^{1}H\}$ NMR spectrum of **10** (50 MHz, $CDCl_3$).

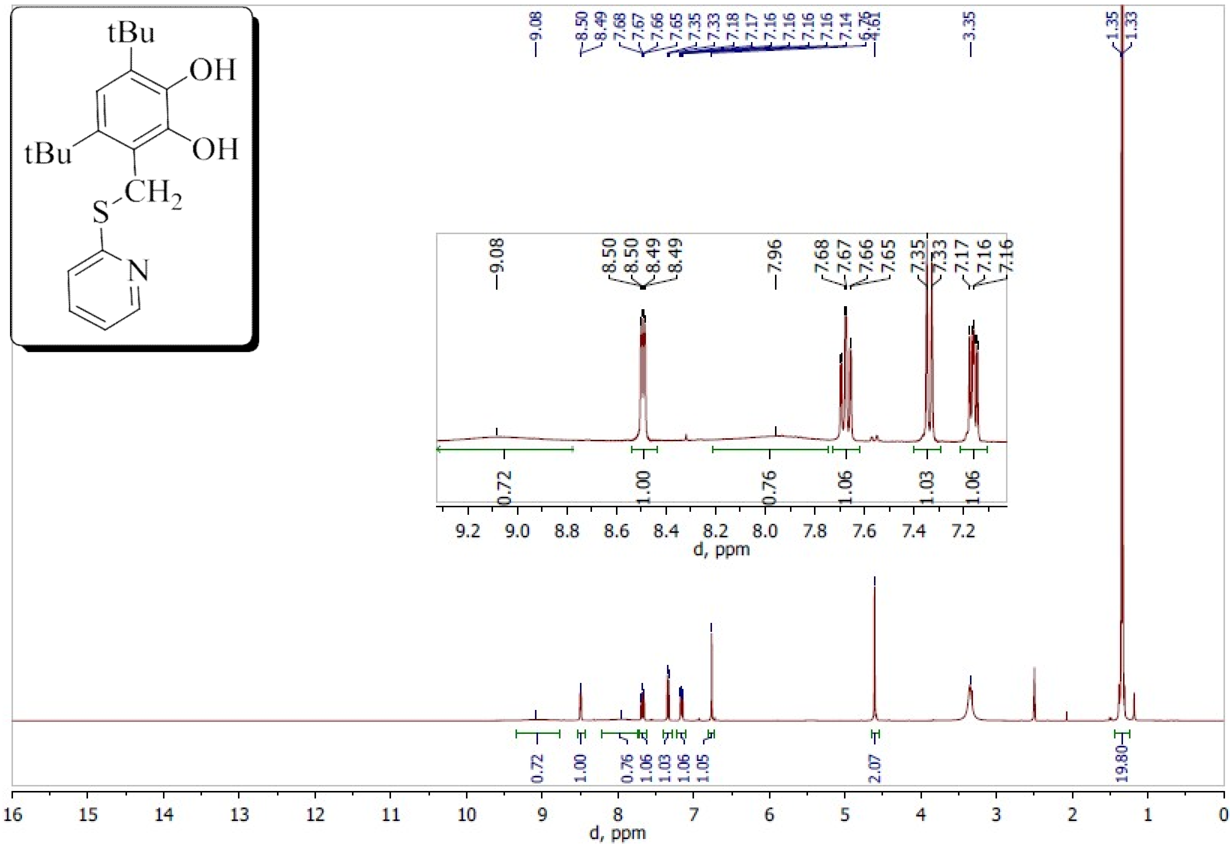

Figure S19. T he ^{1}H NM R spectrum of **11** (400 MHz, DMSO-d$_6$).

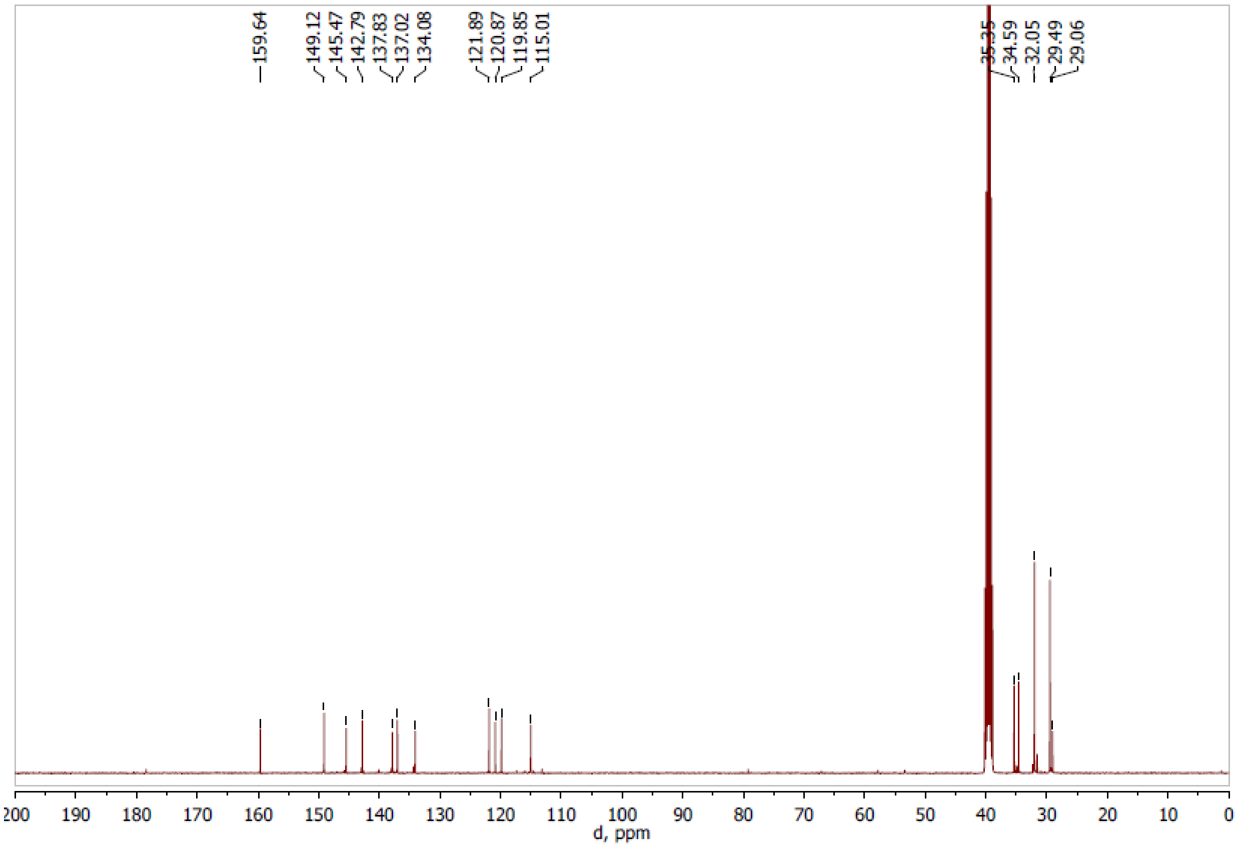

Figure S20. The ^{13}C{^{1}H} NMR spectrum of **11** (100 MHz, DMSO-d$_6$).

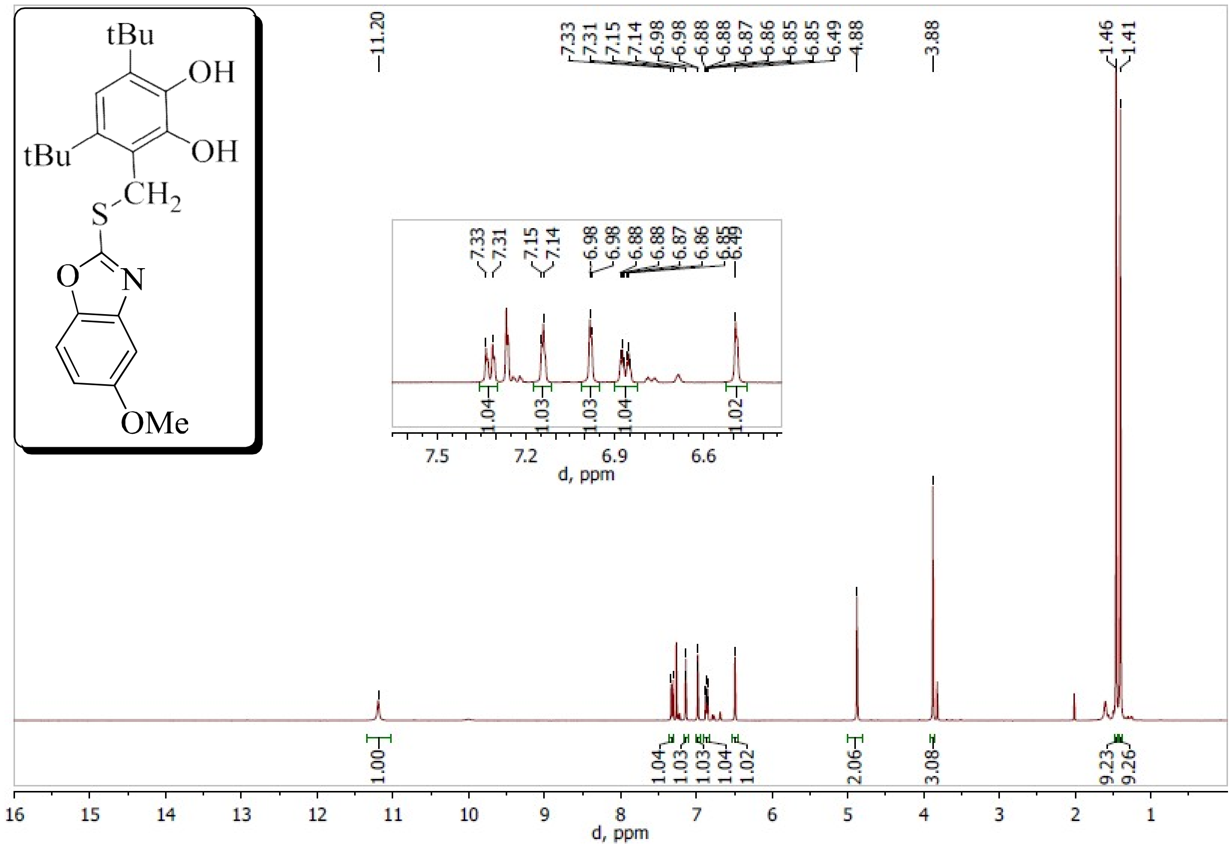

Figure S21. T he ^{1}H NMR spectrum of **12** (400 MHz, $CDCl_3$).

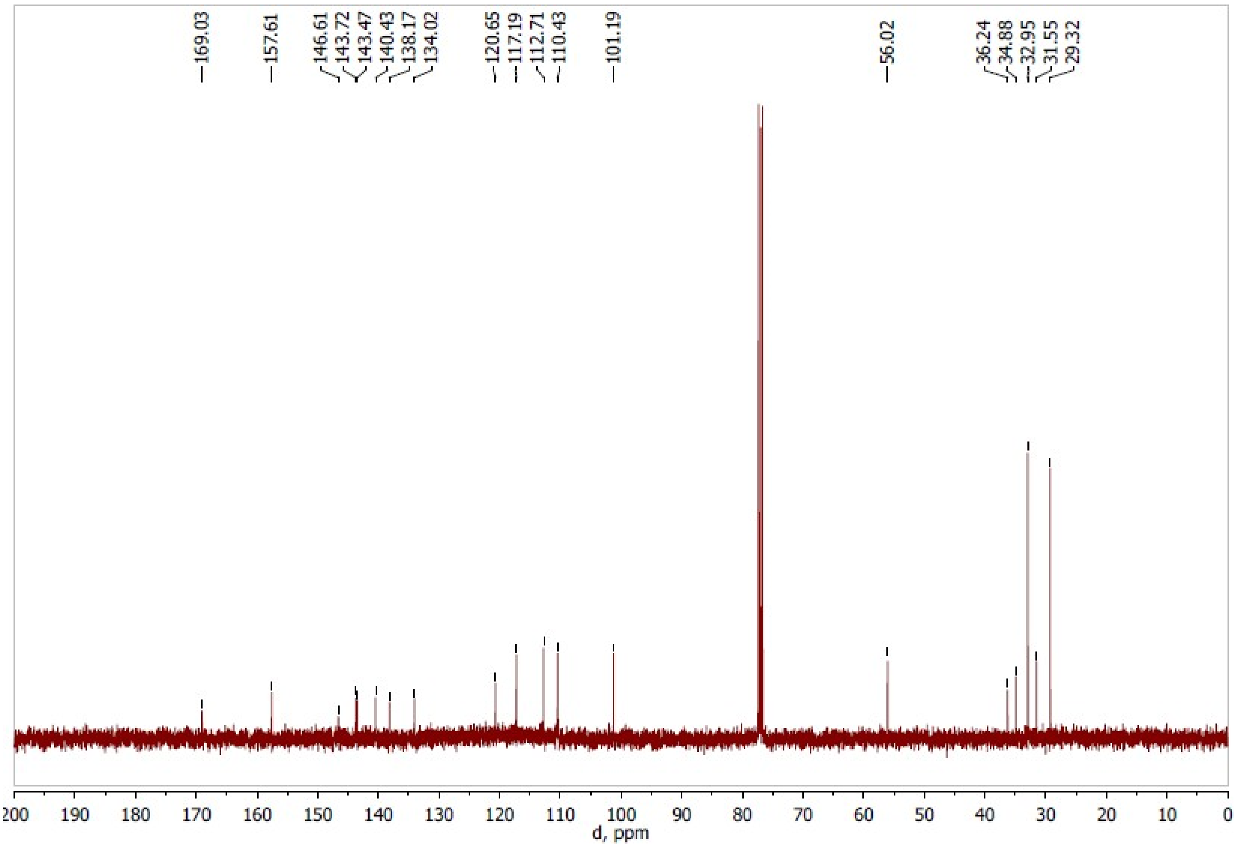

Figure S22. The $^{13}C\{^{1}H\}$ NMR spectrum of **12** (100 MHz, $CDCl_3$).

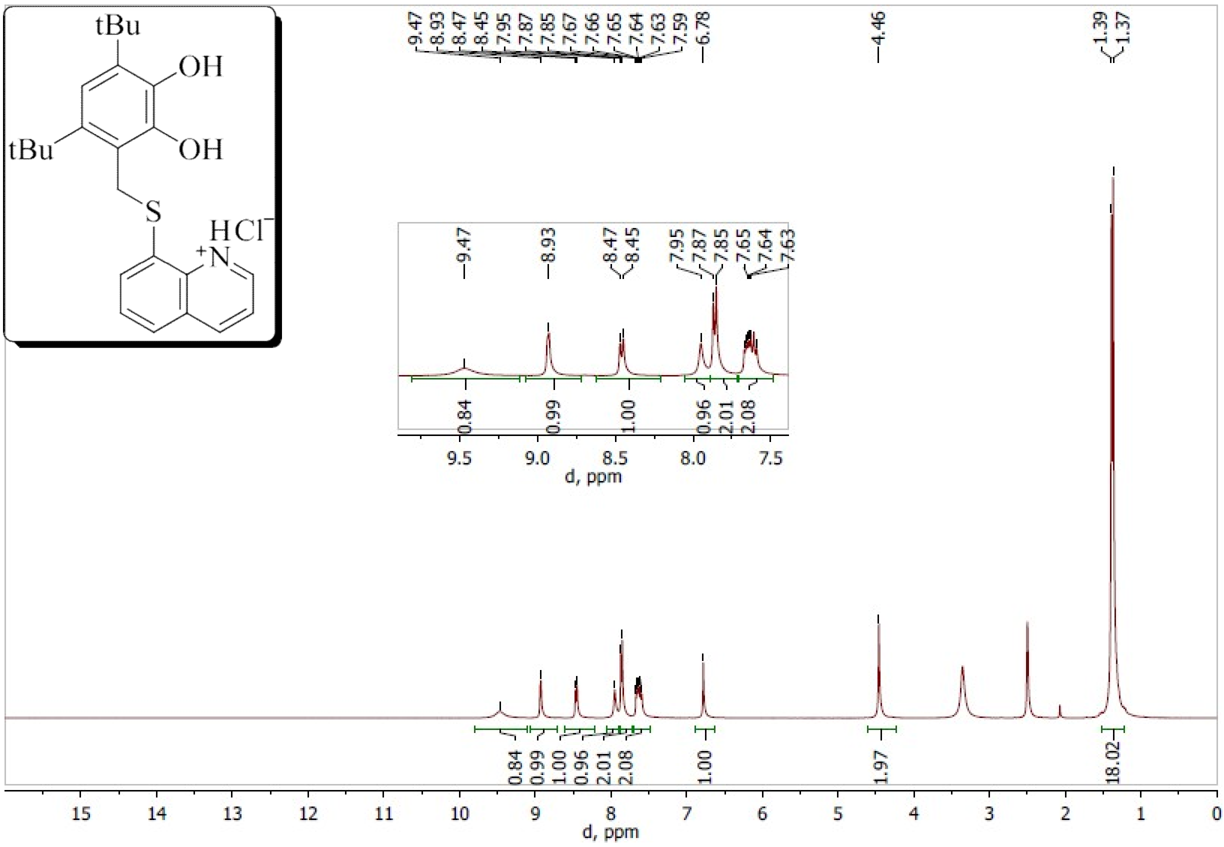

Figure S23. T he ^{1}H NM R spectrum of **13** (400 MHz, DMSO-d_6).

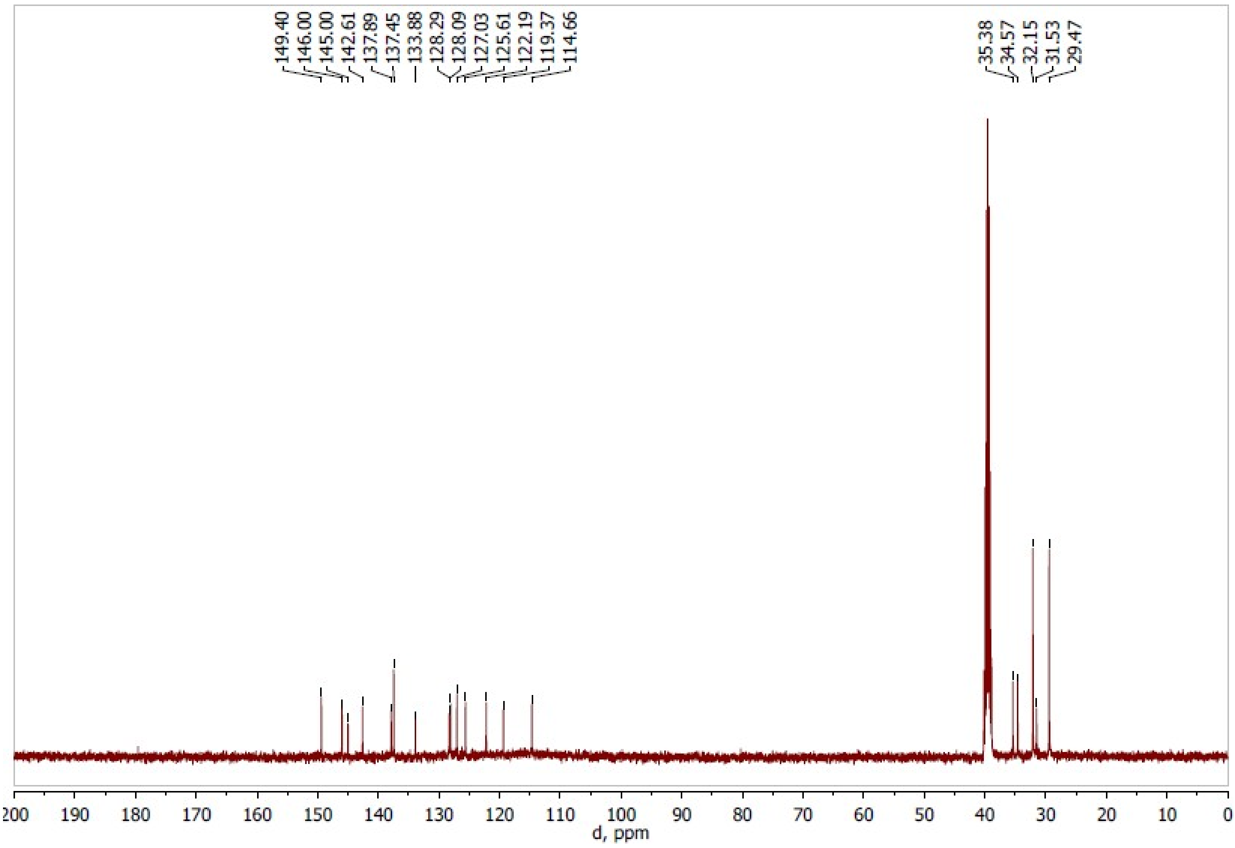

Figure S24. The ^{13}C{^{1}H} NMR spectrum of **13** (100 MHz, DMSO-d_6).

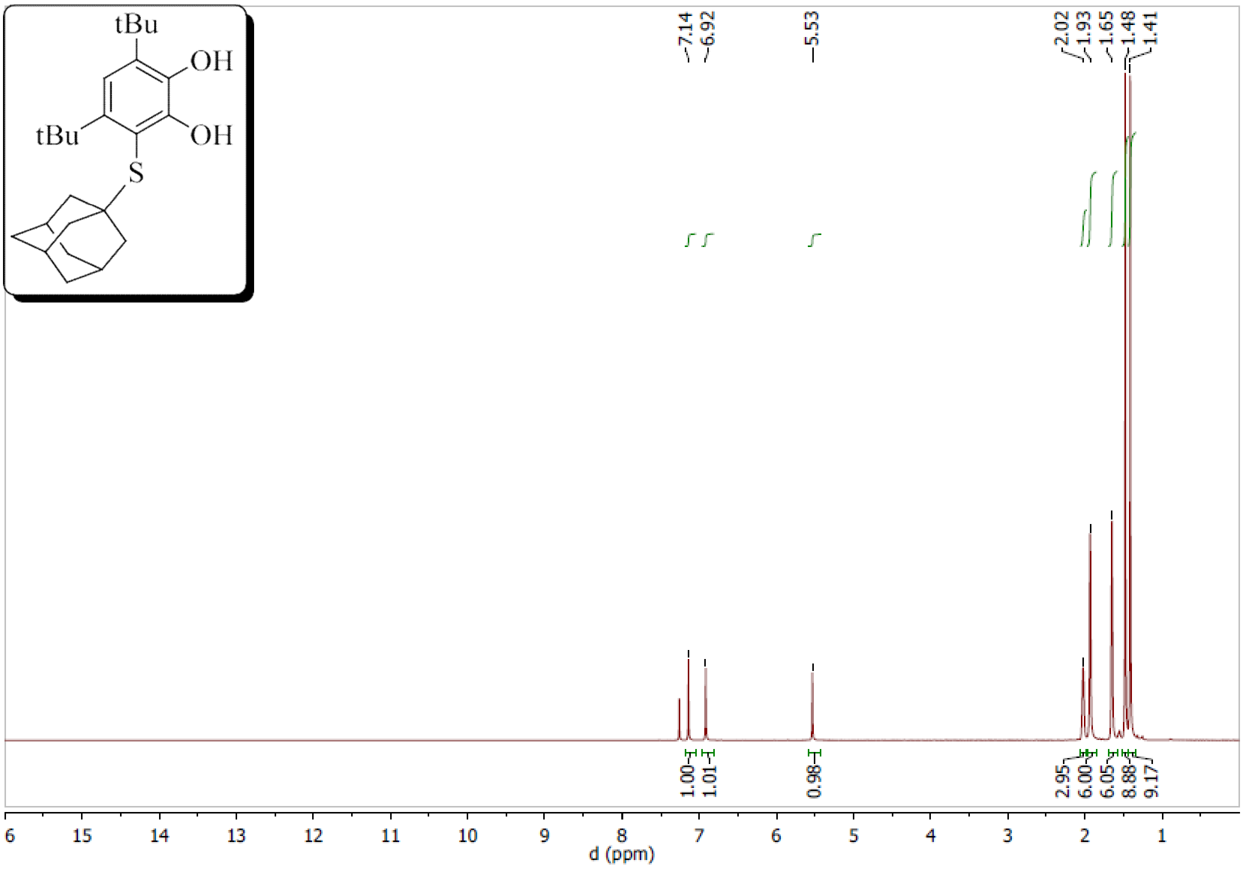

Figure S25. T he ^{1}H NMR spectrum of **14** (400 MHz, $CDCl_3$).

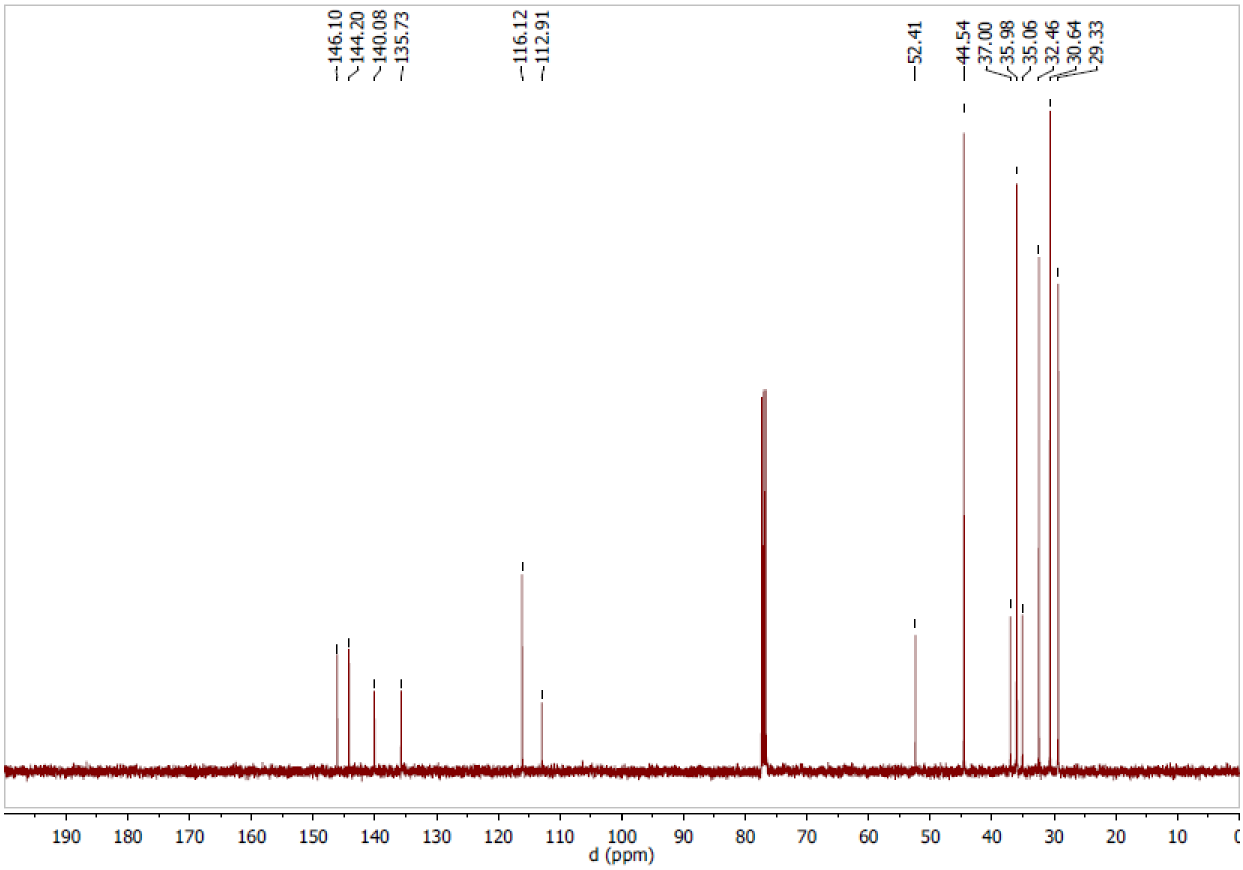

Figure S26. The ^{13}C{^{1}H} NMR spectrum of **14** (100 MHz, $CDCl_3$).

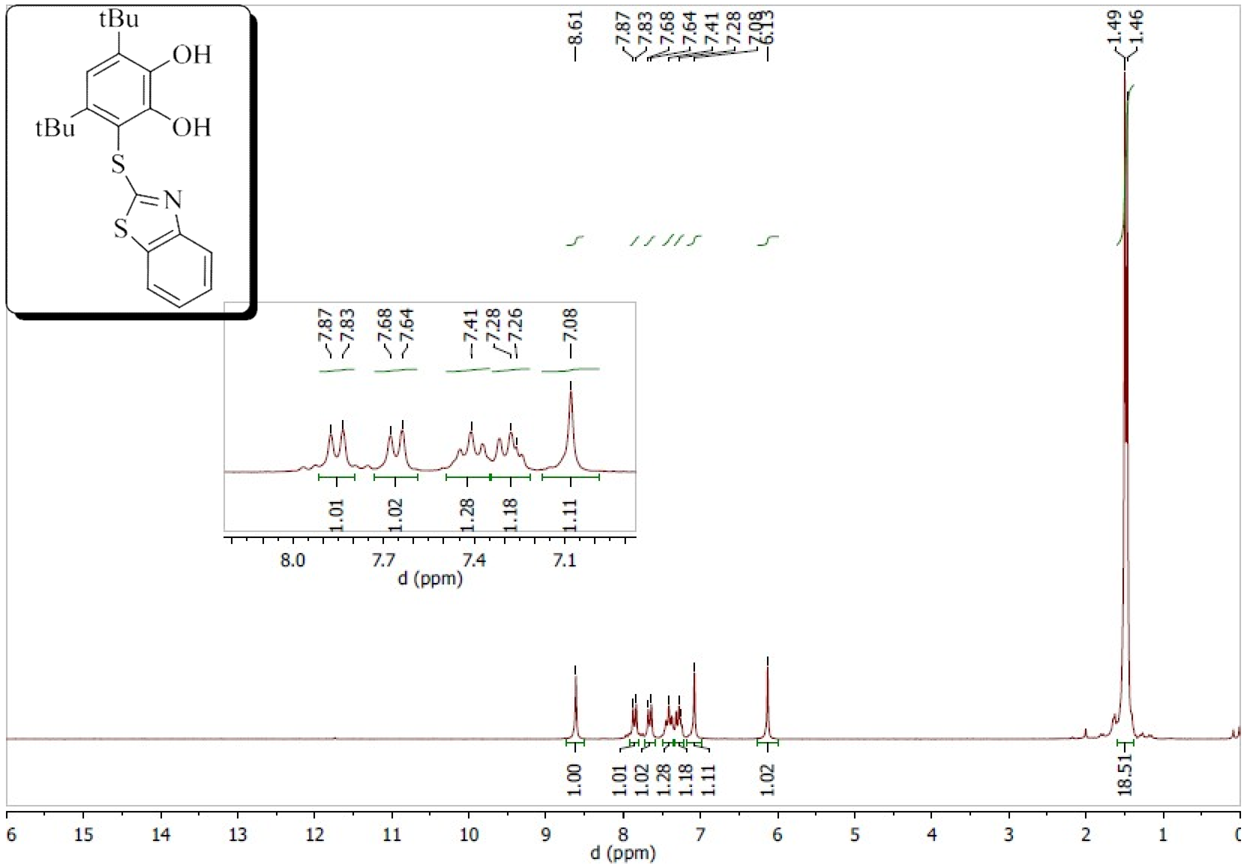

Figure S27. T he ^{1}H NMR spectrum of **15** (400 MHz, $CDCl_3$).

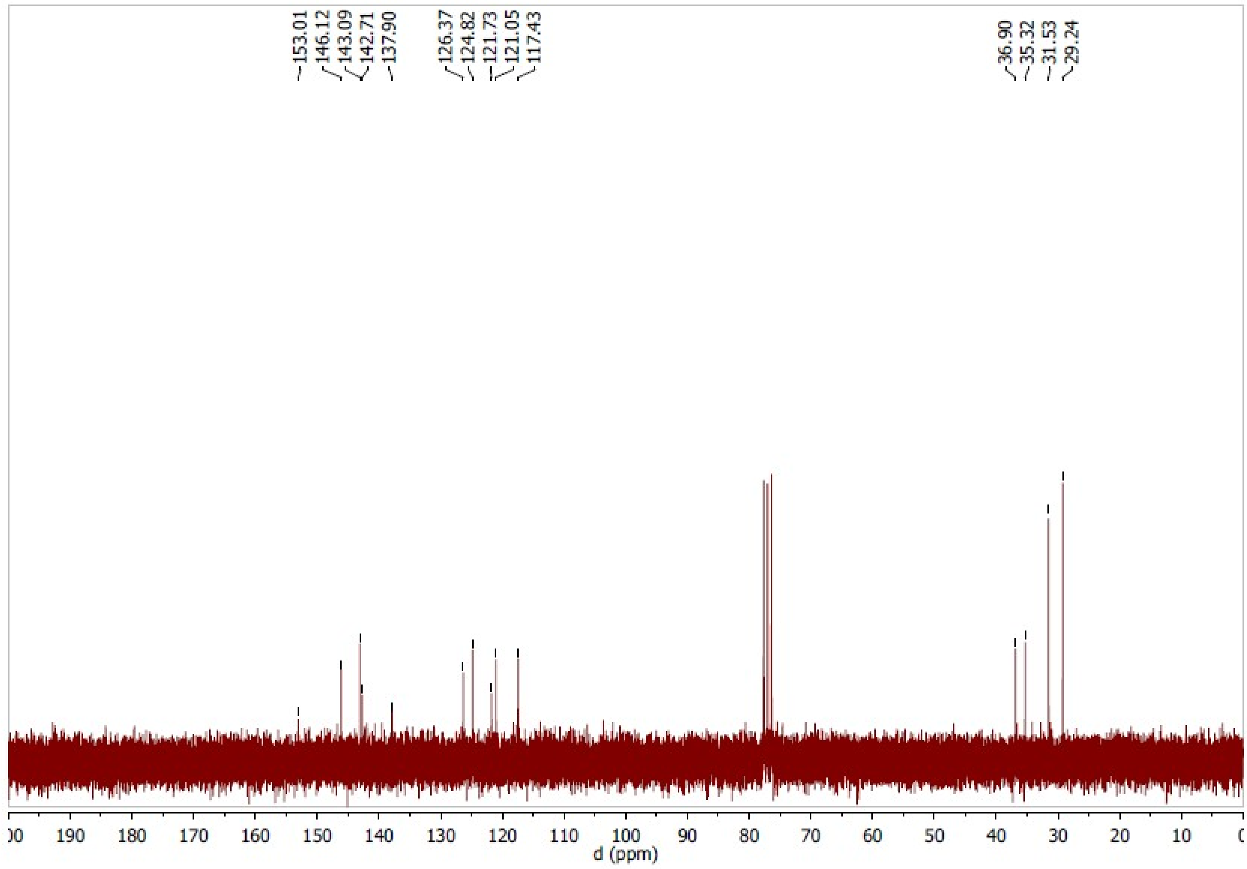

Figure S28. The $^{13}C\{^{1}H\}$ NMR spectrum of **15** (100 MHz, $CDCl_3$).

Table S1. Crystal data and structure refinement for **8** and **15**.

Compound	**8**	**15**
Empirical formula	$C_{22}H_{27}NO_2S_2$	$C_{21}H_{25}NO_2S_2$
Formula weight	401.56	387.54
Temperature, K	298(2)	298(2)
Wavelength, Å	0.71073	
Crystal system	Monoclinic	Triclinic
space group	P2(1)/n	P-1
Unit cell dimensions		
a, Å	9.3517(7)	9.5246(9)
b, Å	15.8237(11)	9.9168(11)
c, Å	15.0865(10)	11.0339(9)
alpha , deg.	90	88.784(8)
beta, deg.	106.800(2)	87.994(7)
gamma, deg.	90	82.011(9)
Volume, $Å^3$	2137.2(3)	1031.30(17)
Z	4	2
Calculated density, Mg/m^3	1.248	1.248
Absorption coefficient, mm^{-1}	0.265	0.273
F(000)	856	412
Crystal size, mm	0.33 x 0.17 x 0.12	0.36 x 0.24 x 0.15
Theta range for data collection, deg.	2.304 - 26.022	2.888 - 26.021
Limiting indices	$-11 \leq h \leq 11$<br>$-19 \leq k \leq 19$<br>$-18 \leq l \leq 18$	$-11 \leq h \leq 11$<br>$-12 \leq k \leq 12$<br>$-13 \leq l \leq 13$
Reflections collected / unique	28454 / 4210 [R(int) = 0.0477]	17741/4059 [R(int) = 0.1186]
Completeness to theta = 25.242, %	99.8	99.8
Absorption correction	Semi-empirical from equivalents	Analytical
Max. and min. transmission	0.9582 and 0.8453	0.964 and 0.927
Refinement method	Full-matrix least-squares on F^2	Full-matrix least-squares on F^2
Data / restraints / parameters	4210 / 0 / 258	4059 / 0 / 243
Goodness-of-fit on F^2	1.070	1.039
Final R indices [I>2sigma(I)]	R1 = 0.0468, wR2 = 0.1236	R1 = 0.0522, wR2 = 0.1237
R indices (all data)	R1 = 0.0625, wR2 = 0.1321	R1 = 0.0677, wR2 = 0.1354
Largest diff. peak and hole, $e{\cdot}Å^{-3}$	0.225 and -0.271	0.314 and -0.331
